# Search for new phenomena with the $$M_{\mathrm {T2}}$$ variable in the all-hadronic final state produced in proton–proton collisions at $$\sqrt{s} = 13$$$$\,\text {TeV}$$

**DOI:** 10.1140/epjc/s10052-017-5267-x

**Published:** 2017-10-26

**Authors:** A. M. Sirunyan, A. Tumasyan, W. Adam, F. Ambrogi, E. Asilar, T. Bergauer, J. Brandstetter, E. Brondolin, M. Dragicevic, J. Erö, M. Flechl, M. Friedl, R. Frühwirth, V. M. Ghete, J. Grossmann, J. Hrubec, M. Jeitler, A. König, N. Krammer, I. Krätschmer, D. Liko, T. Madlener, I. Mikulec, E. Pree, D. Rabady, N. Rad, H. Rohringer, J. Schieck, R. Schöfbeck, M. Spanring, D. Spitzbart, J. Strauss, W. Waltenberger, J. Wittmann, C.-E. Wulz, M. Zarucki, V. Chekhovsky, V. Mossolov, J. Suarez Gonzalez, E. A. De Wolf, D. Di Croce, X. Janssen, J. Lauwers, M. Van De Klundert, H. Van Haevermaet, P. Van Mechelen, N. Van Remortel, A. Van Spilbeeck, S. Abu Zeid, F. Blekman, J. D’Hondt, I. De Bruyn, J. De Clercq, K. Deroover, G. Flouris, D. Lontkovskyi, S. Lowette, S. Moortgat, L. Moreels, A. Olbrechts, Q. Python, K. Skovpen, S. Tavernier, W. Van Doninck, P. Van Mulders, I. Van Parijs, H. Brun, B. Clerbaux, G. De Lentdecker, H. Delannoy, G. Fasanella, L. Favart, R. Goldouzian, A. Grebenyuk, G. Karapostoli, T. Lenzi, J. Luetic, T. Maerschalk, A. Marinov, A. Randle-conde, T. Seva, C. Vander Velde, P. Vanlaer, D. Vannerom, R. Yonamine, F. Zenoni, F. Zhang, A. Cimmino, T. Cornelis, D. Dobur, A. Fagot, M. Gul, I. Khvastunov, D. Poyraz, C. Roskas, S. Salva, M. Tytgat, W. Verbeke, N. Zaganidis, H. Bakhshiansohi, O. Bondu, S. Brochet, G. Bruno, A. Caudron, S. De Visscher, C. Delaere, M. Delcourt, B. Francois, A. Giammanco, A. Jafari, M. Komm, G. Krintiras, V. Lemaitre, A. Magitteri, A. Mertens, M. Musich, K. Piotrzkowski, L. Quertenmont, M. Vidal Marono, S. Wertz, N. Beliy, W. L. Aldá Júnior, F. L. Alves, G. A. Alves, L. Brito, M. Correa Martins Junior, C. Hensel, A. Moraes, M. E. Pol, P. Rebello Teles, E. Belchior Batista Das Chagas, W. Carvalho, J. Chinellato, A. Custódio, E. M. Da Costa, G. G. Da Silveira, D. De Jesus Damiao, S. Fonseca De Souza, L. M. Huertas Guativa, H. Malbouisson, M. Melo De Almeida, C. Mora Herrera, L. Mundim, H. Nogima, A. Santoro, A. Sznajder, E. J. Tonelli Manganote, F. Torres Da Silva De Araujo, A. Vilela Pereira, S. Ahuja, C. A. Bernardes, T. R. Fernandez Perez Tomei, E. M. Gregores, P. G. Mercadante, C. S. Moon, S. F. Novaes, Sandra S. Padula, D. Romero Abad, J. C. Ruiz Vargas, A. Aleksandrov, R. Hadjiiska, P. Iaydjiev, M. Misheva, M. Rodozov, M. Shopova, S. Stoykova, G. Sultanov, A. Dimitrov, I. Glushkov, L. Litov, B. Pavlov, P. Petkov, W. Fang, X. Gao, M. Ahmad, J. G. Bian, G. M. Chen, H. S. Chen, M. Chen, Y. Chen, C. H. Jiang, D. Leggat, Z. Liu, F. Romeo, S. M. Shaheen, A. Spiezia, J. Tao, C. Wang, Z. Wang, E. Yazgan, H. Zhang, J. Zhao, Y. Ban, G. Chen, Q. Li, S. Liu, Y. Mao, S. J. Qian, D. Wang, Z. Xu, C. Avila, A. Cabrera, L. F. Chaparro Sierra, C. Florez, C. F. González Hernández, J. D. Ruiz Alvarez, B. Courbon, N. Godinovic, D. Lelas, I. Puljak, P. M. Ribeiro Cipriano, T. Sculac, Z. Antunovic, M. Kovac, V. Brigljevic, D. Ferencek, K. Kadija, B. Mesic, T. Susa, M. W. Ather, A. Attikis, G. Mavromanolakis, J. Mousa, C. Nicolaou, F. Ptochos, P. A. Razis, H. Rykaczewski, M. Finger, M. Finger, E. Carrera Jarrin, A. Ellithi Kamel, S. Khalil, A. Mohamed, R. K. Dewanjee, M. Kadastik, L. Perrini, M. Raidal, A. Tiko, C. Veelken, P. Eerola, J. Pekkanen, M. Voutilainen, J. Härkönen, T. Järvinen, V. Karimäki, R. Kinnunen, T. Lampén, K. Lassila-Perini, S. Lehti, T. Lindén, P. Luukka, E. Tuominen, J. Tuominiemi, E. Tuovinen, J. Talvitie, T. Tuuva, M. Besancon, F. Couderc, M. Dejardin, D. Denegri, J. L. Faure, F. Ferri, S. Ganjour, S. Ghosh, A. Givernaud, P. Gras, G. Hamel de Monchenault, P. Jarry, I. Kucher, E. Locci, M. Machet, J. Malcles, G. Negro, J. Rander, A. Rosowsky, M. Ö. Sahin, M. Titov, A. Abdulsalam, I. Antropov, S. Baffioni, F. Beaudette, P. Busson, L. Cadamuro, E. Chapon, C. Charlot, O. Davignon, R. Granier de Cassagnac, M. Jo, S. Lisniak, A. Lobanov, J. Martin Blanco, M. Nguyen, C. Ochando, G. Ortona, P. Paganini, P. Pigard, S. Regnard, R. Salerno, J. B. Sauvan, Y. Sirois, A. G. Stahl Leiton, T. Strebler, Y. Yilmaz, A. Zabi, J.-L. Agram, J. Andrea, A. Aubin, J.-M. Brom, M. Buttignol, E. C. Chabert, N. Chanon, C. Collard, E. Conte, X. Coubez, J.-C. Fontaine, D. Gelé, U. Goerlach, M. Jansová, A.-C. Le Bihan, N. Tonon, P. Van Hove, S. Gadrat, S. Beauceron, C. Bernet, G. Boudoul, R. Chierici, D. Contardo, P. Depasse, H. El Mamouni, J. Fay, L. Finco, S. Gascon, M. Gouzevitch, G. Grenier, B. Ille, F. Lagarde, I. B. Laktineh, M. Lethuillier, L. Mirabito, A. L. Pequegnot, S. Perries, A. Popov, V. Sordini, M. Vander Donckt, S. Viret, A. Khvedelidze, Z. Tsamalaidze, C. Autermann, S. Beranek, L. Feld, M. K. Kiesel, K. Klein, M. Lipinski, M. Preuten, C. Schomakers, J. Schulz, T. Verlage, A. Albert, M. Brodski, E. Dietz-Laursonn, D. Duchardt, M. Endres, M. Erdmann, S. Erdweg, T. Esch, R. Fischer, A. Güth, M. Hamer, T. Hebbeker, C. Heidemann, K. Hoepfner, S. Knutzen, M. Merschmeyer, A. Meyer, P. Millet, S. Mukherjee, M. Olschewski, K. Padeken, T. Pook, M. Radziej, H. Reithler, M. Rieger, F. Scheuch, D. Teyssier, S. Thüer, G. Flügge, B. Kargoll, T. Kress, A. Künsken, J. Lingemann, T. Müller, A. Nehrkorn, A. Nowack, C. Pistone, O. Pooth, A. Stahl, M. Aldaya Martin, T. Arndt, C. Asawatangtrakuldee, K. Beernaert, O. Behnke, U. Behrens, A. A. Bin Anuar, K. Borras, V. Botta, A. Campbell, P. Connor, C. Contreras-Campana, F. Costanza, C. Diez Pardos, G. Eckerlin, D. Eckstein, T. Eichhorn, E. Eren, E. Gallo, J. Garay Garcia, A. Geiser, A. Gizhko, J. M. Grados Luyando, A. Grohsjean, P. Gunnellini, A. Harb, J. Hauk, M. Hempel, H. Jung, A. Kalogeropoulos, M. Kasemann, J. Keaveney, C. Kleinwort, I. Korol, D. Krücker, W. Lange, A. Lelek, T. Lenz, J. Leonard, K. Lipka, W. Lohmann, R. Mankel, I.-A. Melzer-Pellmann, A. B. Meyer, G. Mittag, J. Mnich, A. Mussgiller, E. Ntomari, D. Pitzl, R. Placakyte, A. Raspereza, B. Roland, M. Savitskyi, P. Saxena, R. Shevchenko, S. Spannagel, N. Stefaniuk, G. P. Van Onsem, R. Walsh, Y. Wen, K. Wichmann, C. Wissing, O. Zenaiev, S. Bein, V. Blobel, M. Centis Vignali, A. R. Draeger, T. Dreyer, E. Garutti, D. Gonzalez, J. Haller, A. Hinzmann, M. Hoffmann, A. Junkes, A. Karavdina, R. Klanner, R. Kogler, N. Kovalchuk, S. Kurz, T. Lapsien, I. Marchesini, D. Marconi, M. Meyer, M. Niedziela, D. Nowatschin, F. Pantaleo, T. Peiffer, A. Perieanu, C. Scharf, P. Schleper, A. Schmidt, S. Schumann, J. Schwandt, J. Sonneveld, H. Stadie, G. Steinbrück, F. M. Stober, M. Stöver, H. Tholen, D. Troendle, E. Usai, L. Vanelderen, A. Vanhoefer, B. Vormwald, M. Akbiyik, C. Barth, S. Baur, E. Butz, R. Caspart, T. Chwalek, F. Colombo, W. De Boer, A. Dierlamm, B. Freund, R. Friese, M. Giffels, A. Gilbert, D. Haitz, F. Hartmann, S. M. Heindl, U. Husemann, F. Kassel, S. Kudella, H. Mildner, M. U. Mozer, Th. Müller, M. Plagge, G. Quast, K. Rabbertz, M. Schröder, I. Shvetsov, G. Sieber, H. J. Simonis, R. Ulrich, S. Wayand, M. Weber, T. Weiler, S. Williamson, C. Wöhrmann, R. Wolf, G. Anagnostou, G. Daskalakis, T. Geralis, V. A. Giakoumopoulou, A. Kyriakis, D. Loukas, I. Topsis-Giotis, S. Kesisoglou, A. Panagiotou, N. Saoulidou, I. Evangelou, C. Foudas, P. Kokkas, N. Manthos, I. Papadopoulos, E. Paradas, J. Strologas, F. A. Triantis, M. Csanad, N. Filipovic, G. Pasztor, G. Bencze, C. Hajdu, D. Horvath, Á. Hunyadi, F. Sikler, V. Veszpremi, G. Vesztergombi, A. J. Zsigmond, N. Beni, S. Czellar, J. Karancsi, A. Makovec, J. Molnar, Z. Szillasi, M. Bartók, P. Raics, Z. L. Trocsanyi, B. Ujvari, S. Choudhury, J. R. Komaragiri, S. Bahinipati, S. Bhowmik, P. Mal, K. Mandal, A. Nayak, D. K. Sahoo, N. Sahoo, S. K. Swain, S. Bansal, S. B. Beri, V. Bhatnagar, U. Bhawandeep, R. Chawla, N. Dhingra, A. K. Kalsi, A. Kaur, M. Kaur, R. Kumar, P. Kumari, A. Mehta, J. B. Singh, G. Walia, Ashok Kumar, Aashaq Shah, A. Bhardwaj, S. Chauhan, B. C. Choudhary, R. B. Garg, S. Keshri, A. Kumar, S. Malhotra, M. Naimuddin, K. Ranjan, R. Sharma, V. Sharma, R. Bhardwaj, R. Bhattacharya, S. Bhattacharya, S. Dey, S. Dutt, S. Dutta, S. Ghosh, N. Majumdar, A. Modak, K. Mondal, S. Mukhopadhyay, S. Nandan, A. Purohit, A. Roy, D. Roy, S. Roy Chowdhury, S. Sarkar, M. Sharan, S. Thakur, P. K. Behera, R. Chudasama, D. Dutta, V. Jha, V. Kumar, A. K. Mohanty, P. K. Netrakanti, L. M. Pant, P. Shukla, A. Topkar, T. Aziz, S. Dugad, B. Mahakud, S. Mitra, G. B. Mohanty, B. Parida, N. Sur, B. Sutar, S. Banerjee, S. Bhattacharya, S. Chatterjee, P. Das, M. Guchait, Sa. Jain, S. Kumar, M. Maity, G. Majumder, K. Mazumdar, T. Sarkar, N. Wickramage, S. Chauhan, S. Dube, V. Hegde, A. Kapoor, K. Kothekar, S. Pandey, A. Rane, S. Sharma, S. Chenarani, E. Eskandari Tadavani, S. M. Etesami, M. Khakzad, M. Mohammadi Najafabadi, M. Naseri, S. Paktinat Mehdiabadi, F. Rezaei Hosseinabadi, B. Safarzadeh, M. Zeinali, M. Felcini, M. Grunewald, M. Abbrescia, C. Calabria, C. Caputo, A. Colaleo, D. Creanza, L. Cristella, N. De Filippis, M. De Palma, F. Errico, S. Lezki, L. Fiore, G. Iaselli, G. Maggi, M. Maggi, G. Miniello, S. My, S. Nuzzo, A. Pompili, G. Pugliese, R. Radogna, A. Ranieri, G. Selvaggi, A. Sharma, L. Silvestris, R. Venditti, P. Verwilligen, G. Abbiendi, C. Battilana, D. Bonacorsi, S. Braibant-Giacomelli, L. Brigliadori, R. Campanini, P. Capiluppi, A. Castro, F. R. Cavallo, S. S. Chhibra, G. Codispoti, M. Cuffiani, G. M. Dallavalle, F. Fabbri, A. Fanfani, D. Fasanella, P. Giacomelli, L. Guiducci, S. Marcellini, G. Masetti, F. L. Navarria, A. Perrotta, A. M. Rossi, T. Rovelli, G. P. Siroli, N. Tosi, S. Albergo, S. Costa, A. Di Mattia, F. Giordano, R. Potenza, A. Tricomi, C. Tuve, G. Barbagli, K. Chatterjee, V. Ciulli, C. Civinini, R. D’Alessandro, E. Focardi, P. Lenzi, M. Meschini, S. Paoletti, L. Russo, G. Sguazzoni, D. Strom, L. Viliani, L. Benussi, S. Bianco, F. Fabbri, D. Piccolo, F. Primavera, V. Calvelli, F. Ferro, E. Robutti, S. Tosi, L. Brianza, F. Brivio, V. Ciriolo, M. E. Dinardo, S. Fiorendi, S. Gennai, A. Ghezzi, P. Govoni, M. Malberti, S. Malvezzi, R. A. Manzoni, D. Menasce, L. Moroni, M. Paganoni, K. Pauwels, D. Pedrini, S. Pigazzini, S. Ragazzi, T. Tabarelli de Fatis, S. Buontempo, N. Cavallo, S. Di Guida, M. Esposito, F. Fabozzi, F. Fienga, A. O. M. Iorio, W. A. Khan, G. Lanza, L. Lista, S. Meola, P. Paolucci, C. Sciacca, F. Thyssen, P. Azzi, N. Bacchetta, L. Benato, M. Biasotto, D. Bisello, A. Boletti, R. Carlin, A. Carvalho Antunes De Oliveira, P. Checchia, M. Dall’Osso, P. De Castro Manzano, T. Dorigo, U. Dosselli, S. Fantinel, F. Fanzago, U. Gasparini, S. Lacaprara, M. Margoni, A. T. Meneguzzo, N. Pozzobon, P. Ronchese, R. Rossin, F. Simonetto, E. Torassa, M. Zanetti, P. Zotto, A. Braghieri, F. Fallavollita, A. Magnani, P. Montagna, S. P. Ratti, V. Re, M. Ressegotti, C. Riccardi, P. Salvini, I. Vai, P. Vitulo, L. Alunni Solestizi, G. M. Bilei, D. Ciangottini, L. Fanò, P. Lariccia, R. Leonardi, G. Mantovani, V. Mariani, M. Menichelli, A. Saha, A. Santocchia, D. Spiga, K. Androsov, P. Azzurri, G. Bagliesi, J. Bernardini, T. Boccali, L. Borrello, R. Castaldi, M. A. Ciocci, R. Dell’Orso, G. Fedi, L. Giannini, A. Giassi, M. T. Grippo, F. Ligabue, T. Lomtadze, E. Manca, G. Mandorli, L. Martini, A. Messineo, F. Palla, A. Rizzi, A. Savoy-Navarro, P. Spagnolo, R. Tenchini, G. Tonelli, A. Venturi, P. G. Verdini, L. Barone, F. Cavallari, M. Cipriani, D. Del Re, M. Diemoz, S. Gelli, E. Longo, F. Margaroli, B. Marzocchi, P. Meridiani, G. Organtini, R. Paramatti, F. Preiato, S. Rahatlou, C. Rovelli, F. Santanastasio, N. Amapane, R. Arcidiacono, S. Argiro, M. Arneodo, N. Bartosik, R. Bellan, C. Biino, N. Cartiglia, F. Cenna, M. Costa, R. Covarelli, A. Degano, N. Demaria, B. Kiani, C. Mariotti, S. Maselli, E. Migliore, V. Monaco, E. Monteil, M. Monteno, M. M. Obertino, L. Pacher, N. Pastrone, M. Pelliccioni, G. L. Pinna Angioni, F. Ravera, A. Romero, M. Ruspa, R. Sacchi, K. Shchelina, V. Sola, A. Solano, A. Staiano, P. Traczyk, S. Belforte, M. Casarsa, F. Cossutti, G. Della Ricca, A. Zanetti, D. H. Kim, G. N. Kim, M. S. Kim, J. Lee, S. Lee, S. W. Lee, Y. D. Oh, S. Sekmen, D. C. Son, Y. C. Yang, A. Lee, H. Kim, D. H. Moon, G. Oh, J. A. Brochero Cifuentes, J. Goh, T. J. Kim, S. Cho, S. Choi, Y. Go, D. Gyun, S. Ha, B. Hong, Y. Jo, Y. Kim, K. Lee, K. S. Lee, S. Lee, J. Lim, S. K. Park, Y. Roh, J. Almond, J. Kim, J. S. Kim, H. Lee, K. Lee, K. Nam, S. B. Oh, B. C. Radburn-Smith, S. h. Seo, U. K. Yang, H. D. Yoo, G. B. Yu, M. Choi, H. Kim, J. H. Kim, J. S. H. Lee, I. C. Park, G. Ryu, Y. Choi, C. Hwang, J. Lee, I. Yu, V. Dudenas, A. Juodagalvis, J. Vaitkus, I. Ahmed, Z. A. Ibrahim, M. A. B. Md Ali, F. Mohamad Idris, W. A. T. Wan Abdullah, M. N. Yusli, Z. Zolkapli, H. Castilla-Valdez, E. De La Cruz-Burelo, I. Heredia-De La Cruz, R. Lopez-Fernandez, J. Mejia Guisao, A. Sanchez-Hernandez, S. Carrillo Moreno, C. Oropeza Barrera, F. Vazquez Valencia, I. Pedraza, H. A. Salazar Ibarguen, C. Uribe Estrada, A. Morelos Pineda, D. Krofcheck, P. H. Butler, A. Ahmad, M. Ahmad, Q. Hassan, H. R. Hoorani, S. Qazi, A. Saddique, M. Shoaib, M. Waqas, H. Bialkowska, M. Bluj, B. Boimska, T. Frueboes, M. Górski, M. Kazana, K. Nawrocki, K. Romanowska-Rybinska, M. Szleper, P. Zalewski, K. Bunkowski, A. Byszuk, K. Doroba, A. Kalinowski, M. Konecki, J. Krolikowski, M. Misiura, M. Olszewski, A. Pyskir, M. Walczak, P. Bargassa, C. Beirão Da Cruz E Silva, B. Calpas, A. Di Francesco, P. Faccioli, M. Gallinaro, J. Hollar, N. Leonardo, L. Lloret Iglesias, M. V. Nemallapudi, J. Seixas, O. Toldaiev, D. Vadruccio, J. Varela, S. Afanasiev, P. Bunin, M. Gavrilenko, I. Golutvin, I. Gorbunov, A. Kamenev, V. Karjavin, A. Lanev, A. Malakhov, V. Matveev, V. Palichik, V. Perelygin, S. Shmatov, S. Shulha, N. Skatchkov, V. Smirnov, N. Voytishin, A. Zarubin, Y. Ivanov, V. Kim, E. Kuznetsova, P. Levchenko, V. Murzin, V. Oreshkin, I. Smirnov, V. Sulimov, L. Uvarov, S. Vavilov, A. Vorobyev, Yu. Andreev, A. Dermenev, S. Gninenko, N. Golubev, A. Karneyeu, M. Kirsanov, N. Krasnikov, A. Pashenkov, D. Tlisov, A. Toropin, V. Epshteyn, V. Gavrilov, N. Lychkovskaya, V. Popov, I. Pozdnyakov, G. Safronov, A. Spiridonov, A. Stepennov, M. Toms, E. Vlasov, A. Zhokin, T. Aushev, A. Bylinkin, M. Chadeeva, O. Markin, P. Parygin, D. Philippov, S. Polikarpov, V. Rusinov, V. Andreev, M. Azarkin, I. Dremin, M. Kirakosyan, A. Terkulov, A. Baskakov, A. Belyaev, E. Boos, M. Dubinin, L. Dudko, A. Ershov, A. Gribushin, V. Klyukhin, O. Kodolova, I. Lokhtin, I. Miagkov, S. Obraztsov, S. Petrushanko, V. Savrin, A. Snigirev, V. Blinov, Y. Skovpen, D. Shtol, I. Azhgirey, I. Bayshev, S. Bitioukov, D. Elumakhov, V. Kachanov, A. Kalinin, D. Konstantinov, V. Krychkine, V. Petrov, R. Ryutin, A. Sobol, S. Troshin, N. Tyurin, A. Uzunian, A. Volkov, P. Adzic, P. Cirkovic, D. Devetak, M. Dordevic, J. Milosevic, V. Rekovic, J. Alcaraz Maestre, M. Barrio Luna, M. Cerrada, N. Colino, B. De La Cruz, A. Delgado Peris, A. Escalante Del Valle, C. Fernandez Bedoya, J. P. Fernández Ramos, J. Flix, M. C. Fouz, P. Garcia-Abia, O. Gonzalez Lopez, S. Goy Lopez, J. M. Hernandez, M. I. Josa, A. Pérez-Calero Yzquierdo, J. Puerta Pelayo, A. Quintario Olmeda, I. Redondo, L. Romero, M. S. Soares, A. Álvarez Fernández, J. F. de Trocóniz, M. Missiroli, D. Moran, J. Cuevas, C. Erice, J. Fernandez Menendez, I. Gonzalez Caballero, J. R. González Fernández, E. Palencia Cortezon, S. Sanchez Cruz, I. Suárez Andrés, P. Vischia, J. M. Vizan Garcia, I. J. Cabrillo, A. Calderon, B. Chazin Quero, E. Curras, M. Fernandez, J. Garcia-Ferrero, G. Gomez, A. Lopez Virto, J. Marco, C. Martinez Rivero, P. Martinez Ruiz del Arbol, F. Matorras, J. Piedra Gomez, T. Rodrigo, A. Ruiz-Jimeno, L. Scodellaro, N. Trevisani, I. Vila, R. Vilar Cortabitarte, D. Abbaneo, E. Auffray, P. Baillon, A. H. Ball, D. Barney, M. Bianco, P. Bloch, A. Bocci, C. Botta, T. Camporesi, R. Castello, M. Cepeda, G. Cerminara, E. Chapon, Y. Chen, D. d’Enterria, A. Dabrowski, V. Daponte, A. David, M. De Gruttola, A. De Roeck, E. Di Marco, M. Dobson, B. Dorney, T. du Pree, M. Dünser, N. Dupont, A. Elliott-Peisert, P. Everaerts, G. Franzoni, J. Fulcher, W. Funk, D. Gigi, K. Gill, F. Glege, D. Gulhan, S. Gundacker, M. Guthoff, P. Harris, J. Hegeman, V. Innocente, P. Janot, O. Karacheban, J. Kieseler, H. Kirschenmann, V. Knünz, A. Kornmayer, M. J. Kortelainen, C. Lange, P. Lecoq, C. Lourenço, M. T. Lucchini, L. Malgeri, M. Mannelli, A. Martelli, F. Meijers, J. A. Merlin, S. Mersi, E. Meschi, P. Milenovic, F. Moortgat, M. Mulders, H. Neugebauer, S. Orfanelli, L. Orsini, L. Pape, E. Perez, M. Peruzzi, A. Petrilli, G. Petrucciani, A. Pfeiffer, M. Pierini, A. Racz, T. Reis, G. Rolandi, M. Rovere, H. Sakulin, C. Schäfer, C. Schwick, M. Seidel, M. Selvaggi, A. Sharma, P. Silva, P. Sphicas, J. Steggemann, M. Stoye, M. Tosi, D. Treille, A. Triossi, A. Tsirou, V. Veckalns, G. I. Veres, M. Verweij, N. Wardle, W. D. Zeuner, W. Bertl, K. Deiters, W. Erdmann, R. Horisberger, Q. Ingram, H. C. Kaestli, D. Kotlinski, U. Langenegger, T. Rohe, S. A. Wiederkehr, F. Bachmair, L. Bäni, P. Berger, L. Bianchini, B. Casal, G. Dissertori, M. Dittmar, M. Donegà, C. Grab, C. Heidegger, D. Hits, J. Hoss, G. Kasieczka, T. Klijnsma, W. Lustermann, B. Mangano, M. Marionneau, M. T. Meinhard, D. Meister, F. Micheli, P. Musella, F. Nessi-Tedaldi, F. Pandolfi, J. Pata, F. Pauss, G. Perrin, L. Perrozzi, M. Quittnat, M. Rossini, M. Schönenberger, L. Shchutska, A. Starodumov, V. R. Tavolaro, K. Theofilatos, M. L. Vesterbacka Olsson, R. Wallny, A. Zagozdzinska, D. H. Zhu, T. K. Aarrestad, C. Amsler, L. Caminada, M. F. Canelli, A. De Cosa, S. Donato, C. Galloni, T. Hreus, B. Kilminster, J. Ngadiuba, D. Pinna, G. Rauco, P. Robmann, D. Salerno, C. Seitz, A. Zucchetta, V. Candelise, T. H. Doan, Sh. Jain, R. Khurana, M. Konyushikhin, C. M. Kuo, W. Lin, A. Pozdnyakov, S. S. Yu, Arun Kumar, P. Chang, Y. Chao, K. F. Chen, P. H. Chen, F. Fiori, W.-S. Hou, Y. Hsiung, Y. F. Liu, R.-S. Lu, M. Miñano Moya, E. Paganis, A. Psallidas, J. f. Tsai, B. Asavapibhop, K. Kovitanggoon, G. Singh, N. Srimanobhas, A. Adiguzel, F. Boran, S. Cerci, S. Damarseckin, Z. S. Demiroglu, C. Dozen, I. Dumanoglu, S. Girgis, G. Gokbulut, Y. Guler, I. Hos, E. E. Kangal, O. Kara, A. Kayis Topaksu, U. Kiminsu, M. Oglakci, G. Onengut, K. Ozdemir, D. Sunar Cerci, H. Topakli, S. Turkcapar, I. S. Zorbakir, C. Zorbilmez, B. Bilin, G. Karapinar, K. Ocalan, M. Yalvac, M. Zeyrek, E. Gülmez, M. Kaya, O. Kaya, S. Tekten, E. A. Yetkin, M. N. Agaras, S. Atay, A. Cakir, K. Cankocak, B. Grynyov, L. Levchuk, P. Sorokin, R. Aggleton, F. Ball, L. Beck, J. J. Brooke, D. Burns, E. Clement, D. Cussans, H. Flacher, J. Goldstein, M. Grimes, G. P. Heath, H. F. Heath, J. Jacob, L. Kreczko, C. Lucas, D. M. Newbold, S. Paramesvaran, A. Poll, T. Sakuma, S. Seif El Nasr-storey, D. Smith, V. J. Smith, K. W. Bell, A. Belyaev, C. Brew, R. M. Brown, L. Calligaris, D. Cieri, D. J. A. Cockerill, J. A. Coughlan, K. Harder, S. Harper, E. Olaiya, D. Petyt, C. H. Shepherd-Themistocleous, A. Thea, I. R. Tomalin, T. Williams, M. Baber, R. Bainbridge, S. Breeze, O. Buchmuller, A. Bundock, S. Casasso, M. Citron, D. Colling, L. Corpe, P. Dauncey, G. Davies, A. De Wit, M. Della Negra, R. Di Maria, P. Dunne, A. Elwood, D. Futyan, Y. Haddad, G. Hall, G. Iles, T. James, R. Lane, C. Laner, L. Lyons, A.-M. Magnan, S. Malik, L. Mastrolorenzo, T. Matsushita, J. Nash, A. Nikitenko, J. Pela, M. Pesaresi, D. M. Raymond, A. Richards, A. Rose, E. Scott, C. Seez, A. Shtipliyski, S. Summers, A. Tapper, K. Uchida, M. Vazquez Acosta, T. Virdee, D. Winterbottom, J. Wright, S. C. Zenz, J. E. Cole, P. R. Hobson, A. Khan, P. Kyberd, I. D. Reid, P. Symonds, L. Teodorescu, M. Turner, A. Borzou, K. Call, J. Dittmann, K. Hatakeyama, H. Liu, N. Pastika, R. Bartek, A. Dominguez, A. Buccilli, S. I. Cooper, C. Henderson, P. Rumerio, C. West, D. Arcaro, A. Avetisyan, T. Bose, D. Gastler, D. Rankin, C. Richardson, J. Rohlf, L. Sulak, D. Zou, G. Benelli, D. Cutts, A. Garabedian, J. Hakala, U. Heintz, J. M. Hogan, K. H. M. Kwok, E. Laird, G. Landsberg, Z. Mao, M. Narain, S. Piperov, S. Sagir, R. Syarif, D. Yu, R. Band, C. Brainerd, D. Burns, M. Calderon De La Barca Sanchez, M. Chertok, J. Conway, R. Conway, P. T. Cox, R. Erbacher, C. Flores, G. Funk, M. Gardner, W. Ko, R. Lander, C. Mclean, M. Mulhearn, D. Pellett, J. Pilot, S. Shalhout, M. Shi, J. Smith, M. Squires, D. Stolp, K. Tos, M. Tripathi, Z. Wang, M. Bachtis, C. Bravo, R. Cousins, A. Dasgupta, A. Florent, J. Hauser, M. Ignatenko, N. Mccoll, D. Saltzberg, C. Schnaible, V. Valuev, E. Bouvier, K. Burt, R. Clare, J. Ellison, J. W. Gary, S. M. A. Ghiasi Shirazi, G. Hanson, J. Heilman, P. Jandir, E. Kennedy, F. Lacroix, O. R. Long, M. Olmedo Negrete, M. I. Paneva, A. Shrinivas, W. Si, L. Wang, H. Wei, S. Wimpenny, B. R. Yates, J. G. Branson, S. Cittolin, M. Derdzinski, B. Hashemi, A. Holzner, D. Klein, G. Kole, V. Krutelyov, J. Letts, I. Macneill, M. Masciovecchio, D. Olivito, S. Padhi, M. Pieri, M. Sani, V. Sharma, S. Simon, M. Tadel, A. Vartak, S. Wasserbaech, C. Welke, J. Wood, F. Würthwein, A. Yagil, G. Zevi Della Porta, N. Amin, R. Bhandari, J. Bradmiller-Feld, C. Campagnari, A. Dishaw, V. Dutta, M. Franco Sevilla, C. George, F. Golf, L. Gouskos, J. Gran, R. Heller, J. Incandela, S. D. Mullin, A. Ovcharova, H. Qu, J. Richman, D. Stuart, I. Suarez, J. Yoo, D. Anderson, J. Bendavid, A. Bornheim, J. M. Lawhorn, H. B. Newman, T. Nguyen, C. Pena, M. Spiropulu, J. R. Vlimant, S. Xie, Z. Zhang, R. Y. Zhu, M. B. Andrews, T. Ferguson, T. Mudholkar, M. Paulini, J. Russ, M. Sun, H. Vogel, I. Vorobiev, M. Weinberg, J. P. Cumalat, W. T. Ford, F. Jensen, A. Johnson, M. Krohn, S. Leontsinis, T. Mulholland, K. Stenson, S. R. Wagner, J. Alexander, J. Chaves, J. Chu, S. Dittmer, K. Mcdermott, N. Mirman, J. R. Patterson, A. Rinkevicius, A. Ryd, L. Skinnari, L. Soffi, S. M. Tan, Z. Tao, J. Thom, J. Tucker, P. Wittich, M. Zientek, S. Abdullin, M. Albrow, G. Apollinari, A. Apresyan, A. Apyan, S. Banerjee, L. A. T. Bauerdick, A. Beretvas, J. Berryhill, P. C. Bhat, G. Bolla, K. Burkett, J. N. Butler, A. Canepa, G. B. Cerati, H. W. K. Cheung, F. Chlebana, M. Cremonesi, J. Duarte, V. D. Elvira, J. Freeman, Z. Gecse, E. Gottschalk, L. Gray, D. Green, S. Grünendahl, O. Gutsche, R. M. Harris, S. Hasegawa, J. Hirschauer, Z. Hu, B. Jayatilaka, S. Jindariani, M. Johnson, U. Joshi, B. Klima, B. Kreis, S. Lammel, D. Lincoln, R. Lipton, M. Liu, T. Liu, R. Lopes De Sá, J. Lykken, K. Maeshima, N. Magini, J. M. Marraffino, S. Maruyama, D. Mason, P. McBride, P. Merkel, S. Mrenna, S. Nahn, V. O’Dell, K. Pedro, O. Prokofyev, G. Rakness, L. Ristori, B. Schneider, E. Sexton-Kennedy, A. Soha, W. J. Spalding, L. Spiegel, S. Stoynev, J. Strait, N. Strobbe, L. Taylor, S. Tkaczyk, N. V. Tran, L. Uplegger, E. W. Vaandering, C. Vernieri, M. Verzocchi, R. Vidal, M. Wang, H. A. Weber, A. Whitbeck, D. Acosta, P. Avery, P. Bortignon, A. Brinkerhoff, A. Carnes, M. Carver, D. Curry, S. Das, R. D. Field, I. K. Furic, J. Konigsberg, A. Korytov, K. Kotov, P. Ma, K. Matchev, H. Mei, G. Mitselmakher, D. Rank, D. Sperka, N. Terentyev, L. Thomas, J. Wang, S. Wang, J. Yelton, Y. R. Joshi, S. Linn, P. Markowitz, G. Martinez, J. L. Rodriguez, A. Ackert, T. Adams, A. Askew, S. Hagopian, V. Hagopian, K. F. Johnson, T. Kolberg, T. Perry, H. Prosper, A. Santra, R. Yohay, M. M. Baarmand, V. Bhopatkar, S. Colafranceschi, M. Hohlmann, D. Noonan, T. Roy, F. Yumiceva, M. R. Adams, L. Apanasevich, D. Berry, R. R. Betts, R. Cavanaugh, X. Chen, O. Evdokimov, C. E. Gerber, D. A. Hangal, D. J. Hofman, K. Jung, J Kamin, I. D. Sandoval Gonzalez, M. B. Tonjes, H. Trauger, N. Varelas, H. Wang, Z. Wu, M. Zakaria, J. Zhang, B. Bilki, W. Clarida, K. Dilsiz, S. Durgut, R. P. Gandrajula, M. Haytmyradov, V. Khristenko, J.-P. Merlo, H. Mermerkaya, A. Mestvirishvili, A. Moeller, J. Nachtman, H. Ogul, Y. Onel, F. Ozok, A. Penzo, C. Snyder, E. Tiras, J. Wetzel, K. Yi, B. Blumenfeld, A. Cocoros, N. Eminizer, D. Fehling, L. Feng, A. V. Gritsan, P. Maksimovic, J. Roskes, U. Sarica, M. Swartz, M. Xiao, C. You, A. Al-bataineh, P. Baringer, A. Bean, S. Boren, J. Bowen, J. Castle, S. Khalil, A. Kropivnitskaya, D. Majumder, W. Mcbrayer, M. Murray, C. Royon, S. Sanders, E. Schmitz, R. Stringer, J. D. Tapia Takaki, Q. Wang, A. Ivanov, K. Kaadze, Y. Maravin, A. Mohammadi, L. K. Saini, N. Skhirtladze, S. Toda, F. Rebassoo, D. Wright, C. Anelli, A. Baden, O. Baron, A. Belloni, B. Calvert, S. C. Eno, C. Ferraioli, N. J. Hadley, S. Jabeen, G. Y. Jeng, R. G. Kellogg, J. Kunkle, A. C. Mignerey, F. Ricci-Tam, Y. H. Shin, A. Skuja, S. C. Tonwar, D. Abercrombie, B. Allen, V. Azzolini, R. Barbieri, A. Baty, R. Bi, S. Brandt, W. Busza, I. A. Cali, M. D’Alfonso, Z. Demiragli, G. Gomez Ceballos, M. Goncharov, D. Hsu, Y. Iiyama, G. M. Innocenti, M. Klute, D. Kovalskyi, Y. S. Lai, Y.-J. Lee, A. Levin, P. D. Luckey, B. Maier, A. C. Marini, C. Mcginn, C. Mironov, S. Narayanan, X. Niu, C. Paus, C. Roland, G. Roland, J. Salfeld-Nebgen, G. S. F. Stephans, K. Tatar, D. Velicanu, J. Wang, T. W. Wang, B. Wyslouch, A. C. Benvenuti, R. M. Chatterjee, A. Evans, P. Hansen, S. Kalafut, Y. Kubota, Z. Lesko, J. Mans, S. Nourbakhsh, N. Ruckstuhl, R. Rusack, J. Turkewitz, J. G. Acosta, S. Oliveros, E. Avdeeva, K. Bloom, D. R. Claes, C. Fangmeier, R. Gonzalez Suarez, R. Kamalieddin, I. Kravchenko, J. Monroy, J. E. Siado, G. R. Snow, B. Stieger, M. Alyari, J. Dolen, A. Godshalk, C. Harrington, I. Iashvili, D. Nguyen, A. Parker, S. Rappoccio, B. Roozbahani, G. Alverson, E. Barberis, A. Hortiangtham, A. Massironi, D. M. Morse, D. Nash, T. Orimoto, R. Teixeira De Lima, D. Trocino, R.-J. Wang, D. Wood, S. Bhattacharya, O. Charaf, K. A. Hahn, N. Mucia, N. Odell, B. Pollack, M. H. Schmitt, K. Sung, M. Trovato, M. Velasco, N. Dev, M. Hildreth, K. Hurtado Anampa, C. Jessop, D. J. Karmgard, N. Kellams, K. Lannon, N. Loukas, N. Marinelli, F. Meng, C. Mueller, Y. Musienko, M. Planer, A. Reinsvold, R. Ruchti, G. Smith, S. Taroni, M. Wayne, M. Wolf, A. Woodard, J. Alimena, L. Antonelli, B. Bylsma, L. S. Durkin, S. Flowers, B. Francis, A. Hart, C. Hill, W. Ji, B. Liu, W. Luo, D. Puigh, B. L. Winer, H. W. Wulsin, A. Benaglia, S. Cooperstein, P. Elmer, J. Hardenbrook, P. Hebda, S. Higginbotham, D. Lange, J. Luo, D. Marlow, K. Mei, I. Ojalvo, J. Olsen, C. Palmer, P. Piroué, D. Stickland, A. Svyatkovskiy, C. Tully, S. Malik, S. Norberg, A. Barker, V. E. Barnes, S. Folgueras, L. Gutay, M. K. Jha, M. Jones, A. W. Jung, A. Khatiwada, D. H. Miller, N. Neumeister, J. F. Schulte, J. Sun, F. Wang, W. Xie, T. Cheng, N. Parashar, J. Stupak, A. Adair, B. Akgun, Z. Chen, K. M. Ecklund, F. J. M. Geurts, M. Guilbaud, W. Li, B. Michlin, M. Northup, B. P. Padley, J. Roberts, J. Rorie, Z. Tu, J. Zabel, A. Bodek, P. de Barbaro, R. Demina, Y. t. Duh, T. Ferbel, M. Galanti, A. Garcia-Bellido, J. Han, O. Hindrichs, A. Khukhunaishvili, K. H. Lo, P. Tan, M. Verzetti, R. Ciesielski, K. Goulianos, C. Mesropian, A. Agapitos, J. P. Chou, Y. Gershtein, T. A. Gómez Espinosa, E. Halkiadakis, M. Heindl, E. Hughes, S. Kaplan, R. Kunnawalkam Elayavalli, S. Kyriacou, A. Lath, R. Montalvo, K. Nash, M. Osherson, H. Saka, S. Salur, S. Schnetzer, D. Sheffield, S. Somalwar, R. Stone, S. Thomas, P. Thomassen, M. Walker, M. Foerster, J. Heideman, G. Riley, K. Rose, S. Spanier, K. Thapa, O. Bouhali, A. Castaneda Hernandez, A. Celik, M. Dalchenko, M. De Mattia, A. Delgado, S. Dildick, R. Eusebi, J. Gilmore, T. Huang, T. Kamon, R. Mueller, Y. Pakhotin, R. Patel, A. Perloff, L. Perniè, D. Rathjens, A. Safonov, A. Tatarinov, K. A. Ulmer, N. Akchurin, J. Damgov, F. De Guio, P. R. Dudero, J. Faulkner, E. Gurpinar, S. Kunori, K. Lamichhane, S. W. Lee, T. Libeiro, T. Peltola, S. Undleeb, I. Volobouev, Z. Wang, S. Greene, A. Gurrola, R. Janjam, W. Johns, C. Maguire, A. Melo, H. Ni, P. Sheldon, S. Tuo, J. Velkovska, Q. Xu, M. W. Arenton, P. Barria, B. Cox, R. Hirosky, A. Ledovskoy, H. Li, C. Neu, T. Sinthuprasith, X. Sun, Y. Wang, E. Wolfe, F. Xia, C. Clarke, R. Harr, P. E. Karchin, J. Sturdy, S. Zaleski, J. Buchanan, C. Caillol, S. Dasu, L. Dodd, S. Duric, B. Gomber, M. Grothe, M. Herndon, A. Hervé, U. Hussain, P. Klabbers, A. Lanaro, A. Levine, K. Long, R. Loveless, G. A. Pierro, G. Polese, T. Ruggles, A. Savin, N. Smith, W. H. Smith, D. Taylor, N. Woods, CMS Collaboration

**Affiliations:** 1 Yerevan Physics Institute, Yerevan, Armenia; 2 Institut für Hochenergiephysik, Wien, Austria; 3 Institute for Nuclear Problems, Minsk, Belarus; 4 Universiteit Antwerpen, Antwerp, Belgium; 5 Vrije Universiteit Brussel, Brussels, Belgium; 6 Université Libre de Bruxelles, Brussels, Belgium; 7 Ghent University, Ghent, Belgium; 8 Université Catholique de Louvain, Louvain-la-Neuve, Belgium; 9 Université de Mons, Mons, Belgium; 10 Centro Brasileiro de Pesquisas Fisicas, Rio de Janeiro, Brazil; 11 Universidade do Estado do Rio de Janeiro, Rio de Janeiro, Brazil; 12 Universidade Estadual Paulista, Universidade Federal do ABC, São Paulo, Brazil; 13 Institute for Nuclear Research and Nuclear Energy of Bulgaria Academy of Sciences, Sofia, Bulgaria; 14 University of Sofia, Sofia, Bulgaria; 15 Beihang University, Beijing, China; 16 Institute of High Energy Physics, Beijing, China; 17 State Key Laboratory of Nuclear Physics and Technology, Peking University, Beijing, China; 18 Universidad de Los Andes, Bogota, Colombia; 19 Faculty of Electrical Engineering, Mechanical Engineering and Naval Architecture, University of Split, Split, Croatia; 20 Faculty of Science, University of Split, Split, Croatia; 21 Institute Rudjer Boskovic, Zagreb, Croatia; 22 University of Cyprus, Nicosia, Cyprus; 23 Charles University, Prague, Czech Republic; 24 Universidad San Francisco de Quito, Quito, Ecuador; 25 Academy of Scientific Research and Technology of the Arab Republic of Egypt, Egyptian Network of High Energy Physics, Cairo, Egypt; 26 National Institute of Chemical Physics and Biophysics, Tallinn, Estonia; 27 Department of Physics, University of Helsinki, Helsinki, Finland; 28 Helsinki Institute of Physics, Helsinki, Finland; 29 Lappeenranta University of Technology, Lappeenranta, Finland; 30 IRFU, CEA, Université Paris-Saclay, Gif-sur-Yvette, France; 31 Laboratoire Leprince-Ringuet, Ecole Polytechnique, CNRS/IN2P3, Université Paris-Saclay, Palaiseau, France; 32 Université de Strasbourg, CNRS, IPHC UMR 7178, 67000 Strasbourg, France; 33 Centre de Calcul de l’Institut National de Physique Nucleaire et de Physique des Particules, CNRS/IN2P3, Villeurbanne, France; 34 Université de Lyon, Université Claude Bernard Lyon 1, CNRS-IN2P3, Institut de Physique Nucléaire de Lyon, Villeurbanne, France; 35 Georgian Technical University, Tbilisi, Georgia; 36 Tbilisi State University, Tbilisi, Georgia; 37 RWTH Aachen University, I. Physikalisches Institut, Aachen, Germany; 38 RWTH Aachen University, III. Physikalisches Institut A, Aachen, Germany; 39 RWTH Aachen University, III. Physikalisches Institut B, Aachen, Germany; 40 Deutsches Elektronen-Synchrotron, Hamburg, Germany; 41 University of Hamburg, Hamburg, Germany; 42 Institut für Experimentelle Kernphysik, Karlsruhe, Germany; 43 Institute of Nuclear and Particle Physics (INPP), NCSR Demokritos, Aghia Paraskevi, Greece; 44 National and Kapodistrian University of Athens, Athens, Greece; 45 University of Ioánnina, Ioánnina, Greece; 46 MTA-ELTE Lendület CMS Particle and Nuclear Physics Group, Eötvös Loránd University, Budapest, Hungary; 47 Wigner Research Centre for Physics, Budapest, Hungary; 48 Institute of Nuclear Research ATOMKI, Debrecen, Hungary; 49 Institute of Physics, University of Debrecen, Debrecen, Hungary; 50 Indian Institute of Science (IISc), Bangalore, India; 51 National Institute of Science Education and Research, Bhubaneswar, India; 52 Panjab University, Chandigarh, India; 53 University of Delhi, Delhi, India; 54 Saha Institute of Nuclear Physics, HBNI, Kolkata, India; 55 Indian Institute of Technology Madras, Chennai, India; 56 Bhabha Atomic Research Centre, Mumbai, India; 57 Tata Institute of Fundamental Research-A, Mumbai, India; 58 Tata Institute of Fundamental Research-B, Mumbai, India; 59 Indian Institute of Science Education and Research (IISER), Pune, India; 60 Institute for Research in Fundamental Sciences (IPM), Tehran, Iran; 61 University College Dublin, Dublin, Ireland; 62 INFN Sezione di Bari, Università di Bari, Politecnico di Bari, Bari, Italy; 63 INFN Sezione di Bologna, Università di Bologna, Bologna, Italy; 64 INFN Sezione di Catania, Università di Catania, Catania, Italy; 65 INFN Sezione di Firenze, Università di Firenze, Firenze, Italy; 66 INFN Laboratori Nazionali di Frascati, Frascati, Italy; 67 INFN Sezione di Genova, Università di Genova, Genova, Italy; 68 INFN Sezione di Milano-Bicocca, Università di Milano-Bicocca, Milan, Italy; 69 INFN Sezione di Napoli, Università di Napoli ’Federico II’ , Naples, Italy, Università della Basilicata, Potenza, Italy, Università G. Marconi, Rome, Italy; 70 INFN Sezione di Padova, Università di Padova, Padua, Italy, Università di Trento, Trento, Italy; 71 INFN Sezione di Pavia, Università di Pavia, Pavia, Italy; 72 INFN Sezione di Perugia, Università di Perugia, Perugia, Italy; 73 INFN Sezione di Pisa, Università di Pisa, Scuola Normale Superiore di Pisa, Pisa, Italy; 74 INFN Sezione di Roma, Sapienza Università di Roma, Rome, Italy; 75 INFN Sezione di Torino, Università di Torino, Tourin, Italy, Università del Piemonte Orientale, Novara, Italy; 76 INFN Sezione di Trieste, Università di Trieste, Trieste, Italy; 77 Kyungpook National University, Daegu, Korea; 78 Chonbuk National University, Jeonju, Korea; 79 Institute for Universe and Elementary Particles, Chonnam National University, Kwangju, Korea; 80 Hanyang University, Seoul, Korea; 81 Korea University, Seoul, Korea; 82 Seoul National University, Seoul, Korea; 83 University of Seoul, Seoul, Korea; 84 Sungkyunkwan University, Suwon, Korea; 85 Vilnius University, Vilnius, Lithuania; 86 National Centre for Particle Physics, Universiti Malaya, Kuala Lumpur, Malaysia; 87 Centro de Investigacion y de Estudios Avanzados del IPN, Mexico City, Mexico; 88 Universidad Iberoamericana, Mexico City, Mexico; 89 Benemerita Universidad Autonoma de Puebla, Puebla, Mexico; 90 Universidad Autónoma de San Luis Potosí, San Luis Potosí, Mexico; 91 University of Auckland, Auckland, New Zealand; 92 University of Canterbury, Christchurch, New Zealand; 93 National Centre for Physics, Quaid-I-Azam University, Islamabad, Pakistan; 94 National Centre for Nuclear Research, Swierk, Poland; 95 Institute of Experimental Physics, Faculty of Physics, University of Warsaw, Warsaw, Poland; 96 Laboratório de Instrumentação e Física Experimental de Partículas, Lisbon, Portugal; 97 Joint Institute for Nuclear Research, Dubna, Russia; 98 Petersburg Nuclear Physics Institute, Gatchina, St. Petersburg, Russia; 99 Institute for Nuclear Research, Moscow, Russia; 100 Institute for Theoretical and Experimental Physics, Moscow, Russia; 101 Moscow Institute of Physics and Technology, Moscow, Russia; 102 National Research Nuclear University ‘Moscow Engineering Physics Institute’ (MEPhI), Moscow, Russia; 103 P.N. Lebedev Physical Institute, Moscow, Russia; 104 Skobeltsyn Institute of Nuclear Physics, Lomonosov Moscow State University, Moscow, Russia; 105 Novosibirsk State University (NSU), Novosibirsk, Russia; 106 State Research Center of Russian Federation, Institute for High Energy Physics, Protvino, Russia; 107 Faculty of Physics and Vinca Institute of Nuclear Sciences, University of Belgrade, Belgrade, Serbia; 108 Centro de Investigaciones Energéticas Medioambientales y Tecnológicas (CIEMAT), Madrid, Spain; 109 Universidad Autónoma de Madrid, Madrid, Spain; 110 Universidad de Oviedo, Oviedo, Spain; 111 Instituto de Física de Cantabria (IFCA), CSIC-Universidad de Cantabria, Santander, Spain; 112 CERN, European Organization for Nuclear Research, Geneva, Switzerland; 113 Paul Scherrer Institut, Villigen, Switzerland; 114 Institute for Particle Physics, ETH Zurich, Zurich, Switzerland; 115 Universität Zürich, Zurich, Switzerland; 116 National Central University, Chung-Li, Taiwan; 117 National Taiwan University (NTU), Taipei, Taiwan; 118 Department of Physics, Faculty of Science, Chulalongkorn University, Bangkok, Thailand; 119 Cukurova University-Physics Department, Science and Art Faculty, Adana, Turkey; 120 Physics Department, Middle East Technical University, Ankara, Turkey; 121 Bogazici University, Istanbul, Turkey; 122 Istanbul Technical University, Istanbul, Turkey; 123 Institute for Scintillation Materials of National Academy of Science of Ukraine, Kharkov, Ukraine; 124 National Scientific Center, Kharkov Institute of Physics and Technology, Kharkov, Ukraine; 125 University of Bristol, Bristol, UK; 126 Rutherford Appleton Laboratory, Didcot, UK; 127 Imperial College, London, UK; 128 Brunel University, Uxbridge, UK; 129 Baylor University, Waco, USA; 130 Catholic University of America, Washington, DC USA; 131 The University of Alabama, Tuscaloosa, USA; 132 Boston University, Boston, USA; 133 Brown University, Providence, USA; 134 University of California, Davis, CA USA; 135 University of California, Los Angeles, USA; 136 University of California, Riverside, CA USA; 137 University of California, San Diego, La Jolla USA; 138 University of California, Santa Barbara - Department of Physics, Santa Barbara, USA; 139 California Institute of Technology, Pasadena, USA; 140 Carnegie Mellon University, Pittsburgh, USA; 141 University of Colorado Boulder, Boulder, USA; 142 Cornell University, Ithaca, USA; 143 Fermi National Accelerator Laboratory, Batavia, USA; 144 University of Florida, Gainesville, USA; 145 Florida International University, Miami, USA; 146 Florida State University, Tallahassee, USA; 147 Florida Institute of Technology, Melbourne, USA; 148 University of Illinois at Chicago (UIC), Chicago, USA; 149 The University of Iowa, Iowa City, USA; 150 Johns Hopkins University, Baltimore, USA; 151 The University of Kansas, Lawrence, USA; 152 Kansas State University, Manhattan, USA; 153 Lawrence Livermore National Laboratory, Livermore, USA; 154 University of Maryland, College Park, USA; 155 Massachusetts Institute of Technology, Cambridge, USA; 156 University of Minnesota, Minneapolis, USA; 157 University of Mississippi, Oxford, USA; 158 University of Nebraska-Lincoln, Lincoln, USA; 159 State University of New York at Buffalo, Buffalo, USA; 160 Northeastern University, Boston, USA; 161 Northwestern University, Evanston, USA; 162 University of Notre Dame, Notre Dame, USA; 163 The Ohio State University, Columbus, USA; 164 Princeton University, Princeton, USA; 165 University of Puerto Rico, Mayagüez, USA; 166 Purdue University, West Lafayette, USA; 167 Purdue University Northwest, Hammond, USA; 168 Rice University, Houston, USA; 169 University of Rochester, Rochester, USA; 170 The Rockefeller University, New York, USA; 171 Rutgers, The State University of New Jersey, Piscataway, USA; 172 University of Tennessee, Knoxville, USA; 173 Texas A&M University, College Station, USA; 174 Texas Tech University, Lubbock, USA; 175 Vanderbilt University, Nashville, USA; 176 University of Virginia, Charlottesville, USA; 177 Wayne State University, Detroit, USA; 178 University of Wisconsin - Madison, Madison, WI USA; 179 CERN, 1211 Geneva 23, Switzerland

## Abstract

A search for new phenomena is performed using events with jets and significant transverse momentum imbalance, as inferred through the $M_{\mathrm {T2}}$ variable. The results are based on a sample of proton–proton collisions collected in 2016 at a center-of-mass energy of 13$\,\text {TeV}$ with the CMS detector and corresponding to an integrated luminosity of 35.9$\,\text {fb}^\text {-1}$. No excess event yield is observed above the predicted standard model background, and the results are interpreted as exclusion limits at 95% confidence level on the masses of predicted particles in a variety of simplified models of *R*-parity conserving supersymmetry. Depending on the details of the model, 95% confidence level lower limits on the gluino (light-flavor squark) masses are placed up to 2025 (1550)$\,\text {GeV}$. Mass limits as high as 1070 (1175)$\,\text {GeV}$ are set on the masses of top (bottom) squarks. Information is provided to enable re-interpretation of these results, including model-independent limits on the number of non-standard model events for a set of simplified, inclusive search regions.

## Introduction

We present results of a search for new phenomena in events with jets and significant transverse momentum imbalance in proton–proton collisions at $\sqrt{s} = 13\,\text {TeV}$. Such searches were previously conducted by both the ATLAS [1–5] and CMS [6–9] Collaborations. Our search builds on the work presented in Ref. [6], using improved methods to estimate the background from standard model (SM) processes and a data set corresponding to an integrated luminosity of 35.9$\,\text {fb}^\text {-1}$ of pp collisions collected during 2016 with the CMS detector at the CERN LHC. Event counts in bins of the number of jets ($N_{\mathrm {j}}$), the number of b-tagged jets ($N_{\mathrm {b}}$), the scalar sum of the transverse momenta $p_{\mathrm {T}}$ of all selected jets ($H_{\mathrm {T}}$), and the $M_{\mathrm {T2}}$ variable [6, 10] are compared against estimates of the background from SM processes derived from dedicated data control samples. We observe no evidence for a significant excess above the expected background event yield and interpret the results as exclusion limits at 95% confidence level on the production of pairs of gluinos and squarks using simplified models of supersymmetry (SUSY) [11–18]. Model-independent limits on the number of non-SM events are also provided for a simpler set of inclusive search regions.

## The CMS detector

The central feature of the CMS apparatus is a superconducting solenoid of 6$\text {\,m}$ internal diameter, providing a magnetic field of 3.8$\text {\,T}$. Within the solenoid volume are a silicon pixel and strip tracker, a lead tungstate crystal electromagnetic calorimeter, and a brass and scintillator hadron calorimeter, each composed of a barrel and two endcap sections. Forward calorimeters extend the pseudorapidity ($\eta$) coverage provided by the barrel and endcap detectors. Muons are measured in gas-ionization detectors embedded in the steel flux-return yoke outside the solenoid. The first level of the CMS trigger system, composed of custom hardware processors, uses information from the calorimeters and muon detectors to select the most interesting events in a fixed time interval of less than 4$\,\mu \text {s}$. The high-level trigger processor farm further decreases the event rate from around 100$\text {\,kHz}$ to less than 1$\text {\,kHz}$, before data storage. A more detailed description of the CMS detector and trigger system, together with a definition of the coordinate system used and the relevant kinematic variables, can be found in Refs. [19, 20].

## Event selection and Monte Carlo simulation

Events are processed using the particle-flow (PF) algorithm [21], which is designed to reconstruct and identify all particles using the optimal combination of information from the elements of the CMS detector. Physics objects reconstructed with this algorithm are hereafter referred to as particle-flow candidates. The physics objects and the event preselection are similar to those described in Ref. [6], and are summarized in Table 1. We select events with at least one jet, and veto events with an isolated lepton ($\mathrm {e}$ or $\mu$) or charged PF candidate. The isolated charged PF candidate selection is designed to provide additional rejection against events with electrons and muons, as well as to reject hadronic tau decays. Jets are formed by clustering PF candidates using the anti-$k_{\mathrm {T}}$ algorithm [22, 23] and are corrected for contributions from event pileup [24] and the effects of non-uniform detector response. Only jets passing the selection criteria in Table 1 are used for counting and the determination of kinematic variables. Jets consistent with originating from a heavy-flavor hadron are identified using the combined secondary vertex tagging algorithm [25], with a working point chosen such that the efficiency to identify a $\mathrm {b}$ quark jet is in the range 50–65% for jet $p_{\mathrm {T}}$ between 20 and 400$\,\text {GeV}$. The misidentification rate is approximately 1% for light-flavor and gluon jets and 10% for charm jets. A more detailed discussion of the algorithm performance is given in Ref. [25].

The negative of the vector sum of the $p_{\mathrm {T}}$ of all selected jets is denoted by $\vec {H}_{\mathrm {T}}^{\text {miss}}$, while ${\vec p}_{\mathrm {T}}^{\text {miss}}$ is defined as the negative of the vector $p_{\mathrm {T}}$ sum of all reconstructed PF candidates. The jet corrections are also used to correct ${\vec p}_{\mathrm {T}}^{\text {miss}}$. Events with possible contributions from beam-halo processes or anomalous noise in the calorimeter are rejected using dedicated filters [26, 27]. For events with at least two jets, we start with the pair having the largest dijet invariant mass and iteratively cluster all selected jets using a hemisphere algorithm that minimizes the Lund distance measure [28, 29] until two stable pseudo-jets are obtained. The resulting pseudo-jets together with the ${\vec p}_{\mathrm {T}}^{\text {miss}}$ are used to calculate the kinematic variable $M_{\mathrm {T2}}$ as:

1 $$\begin{aligned} M_{\mathrm {T2}} = \min _{{\vec p}_{\mathrm {T}}^{\text {miss}} {}^{ \mathrm {X}(1)} + {\vec p}_{\mathrm {T}}^{\text {miss}} {}^{ \mathrm {X}(2)} = {\vec p}_{\mathrm {T}}^{\text {miss}}} \left[ \max \left( M_{\mathrm {T}} ^{(1)} , M_{\mathrm {T}} ^{(2)} \right) \right] , \end{aligned}$$

where ${\vec p}_{\mathrm {T}}^{\text {miss}} {}^{ \mathrm {X}(i)}$ ($i=1$,2) are trial vectors obtained by decomposing ${\vec p}_{\mathrm {T}}^{\text {miss}}$, and $M_{\mathrm {T}} ^{(i)}$ are the transverse masses obtained by pairing either of the trial vectors with one of the two pseudo-jets. The minimization is performed over all trial momenta satisfying the ${\vec p}_{\mathrm {T}}^{\text {miss}}$ constraint. The background from multijet events (discussed in Sect. 4) is characterized by small values of $M_{\mathrm {T2}}$, while larger $M_{\mathrm {T2}}$ values are obtained in processes with significant, genuine ${\vec p}_{\mathrm {T}}^{\text {miss}}$.

**Table 1:** Summary of reconstruction objects and event preselection. Here *R* is the distance parameter of the anti-$k_{\mathrm {T}}$ algorithm. For veto leptons and tracks, the transverse mass $M_{\mathrm {T}}$ is determined using the veto object and the ${\vec p}_{\mathrm {T}}^{\text {miss}}$. The variable $p_{\mathrm {T}} ^{\text {sum}}$ is a measure of isolation and it denotes the sum of the transverse momenta of all the PF candidates in a cone around the lepton or the track. The size of the cone, in units of $\varDelta R \equiv \sqrt{\smash [b]{(\varDelta \phi )^2 + (\varDelta \eta )^2}}$ is given in the table. Further details of the lepton selection are described in Ref. [6]. The *i*th highest-$p_{\mathrm {T}}$ jet is denoted as $\hbox {j}_i$

Trigger	$p_{\mathrm {T}} ^\text {miss} >120\,\text {GeV}$ and $H_{\mathrm {T}}^{\mathrm {miss}} >120\,\text {GeV}$ or
	$H_{\mathrm {T}} >300\,\text {GeV}$ and $p_{\mathrm {T}} ^\text {miss} >110\,\text {GeV}$ or
	$H_{\mathrm {T}} >900\,\text {GeV}$ or jet $p_{\mathrm {T}} >450\,\text {GeV}$
Jet selection	$R=0.4$, $p_{\mathrm {T}} >30\,\text {GeV}$, $|\eta |<2.4$
$\mathrm {b}$ tag selection	$p_{\mathrm {T}} >20\,\text {GeV}$, $|\eta |<2.4$
$p_{\mathrm {T}} ^\text {miss}$	$p_{\mathrm {T}} ^\text {miss} >250\,\text {GeV}$ for $H_{\mathrm {T}} <1000\,\text {GeV}$, else $p_{\mathrm {T}} ^\text {miss} >30\,\text {GeV}$
	$$\varDelta \phi _{\text {min}} = \varDelta \phi \left( p_{\mathrm {T}} ^\text {miss}, j_{\mathrm {1,2,3,4}}\right) >0.3$$
	$$|{\vec p}_{\mathrm {T}}^{\text {miss}}-\vec {H}_{\mathrm {T}}^{\text {miss}} |/p_{\mathrm {T}} ^\text {miss} <0.5$$
$M_{\mathrm {T2}}$	$M_{\mathrm {T2}} >200\,\text {GeV}$ for $H_{\mathrm {T}} <1500\,\text {GeV}$, else$M_{\mathrm {T2}} >400\,\text {GeV}$
Veto muon	$p_{\mathrm {T}} >10\,\text {GeV}$, $|\eta |<2.4$, $p_{\mathrm {T}} ^{\text {sum}} < 0.2 \,p_{\mathrm {T}} ^{\text {lep}}$ or
	$p_{\mathrm {T}} >5\,\text {GeV}$, $|\eta |<2.4$, $M_{\mathrm {T}} <100\,\text {GeV}$, $p_{\mathrm {T}} ^{\text {sum}}< 0.2 \, p_{\mathrm {T}} ^{\text {lep}}$
Veto electron	$p_{\mathrm {T}} >10\,\text {GeV}$, $|\eta |<2.4$, $p_{\mathrm {T}} ^{\text {sum}} < 0.1 \, p_{\mathrm {T}} ^{\text {lep}}$ or
	$p_{\mathrm {T}} >5\,\text {GeV}$, $|\eta |<2.4$, $M_{\mathrm {T}} <100\,\text {GeV}$, $p_{\mathrm {T}} ^{\text {sum}}< 0.2 \, p_{\mathrm {T}} ^{\text {lep}}$
Veto track	$p_{\mathrm {T}} >10\,\text {GeV}$, $|\eta |<2.4$, $M_{\mathrm {T}} <100\,\text {GeV}$,$p_{\mathrm {T}} ^{\text {sum}} < 0.1 \, p_{\mathrm {T}} ^{\text {track}}$
$p_{\mathrm {T}} ^{\text {sum}}$ cone	Veto e or $\mathrm {\mu }$: $\varDelta R= \min (0.2, \max (10\,\text {GeV}/p_{\mathrm {T}} ^{\text {lep}},0.05))$
	Veto track: $\varDelta R=$ 0.3

Collision events are selected using triggers with requirements on $H_{\mathrm {T}}$, $p_{\mathrm {T}} ^\text {miss}$, $H_{\mathrm {T}}^{\mathrm {miss}}$, and jet $p_{\mathrm {T}}$. The combined trigger efficiency, as measured in a data sample of events with an isolated electron, is found to be >98% across the full kinematic range of the search. To suppress background from multijet production, we require $M_{\mathrm {T2}}>$ 200$\,\text {GeV}$ in events with $N_{\mathrm {j}} \ge 2$ and $H_{\mathrm {T}} < 1500$ $\,\text {GeV}$. This $M_{\mathrm {T2}}$ threshold is increased to 400$\,\text {GeV}$ for events with $H_{\mathrm {T}} > 1500$ $\,\text {GeV}$ to maintain multijet processes as a subdominant background in all search regions. To protect against jet mismeasurement, we require the minimum difference in azimuthal angle between the ${\vec p}_{\mathrm{T}}^{\text{miss}}$ vector and each of the leading four jets, $\varDelta \phi _{\text{min}}$, to be greater than 0.3, and the magnitude of the difference between ${\vec p}_{\mathrm{T}}^{\text{miss}}$ and $\vec {H}_{\mathrm{T}}^{\text{miss}}$ to be less than half of $p_{\mathrm {T}} ^\text{miss}$. For the determination of $\varDelta \phi _{\text{min}}$ we consider jets with $|\eta |<4.7$. If less than four such jets are found, all are considered in the $\varDelta \phi _{\text{min}}$ calculation.

Events containing at least two jets are categorized by the values of $N_{\mathrm{j}}$, $N_{\mathrm{b}}$, and $H_{\mathrm{T}}$. Each such bin is referred to as a *topological region*. Signal regions are defined by further dividing topological regions into bins of $M_{\mathrm{T2}}$. Events with only one jet are selected if the $p_{\mathrm{T}}$ of the jet is at least 250$\,\text {GeV}$, and are classified according to the $p_{\mathrm{T}}$ of this jet and whether the event contains a b-tagged jet. The search regions are summarized in Tables 5, 6, 7 in Appendix A. We also define *super signal regions*, covering a subset of the kinematic space of the full analysis with simpler inclusive selections. The super signal regions can be used to obtain approximate interpretations of our result, as discussed in Sect. 5, where these regions are defined.

Monte Carlo (MC) simulations are used to design the search, to aid in the estimation of SM backgrounds, and to evaluate the sensitivity to gluino and squark pair production in simplified models of SUSY. The main background samples ($\text {Z+jets}$, $\text {W+jets}$, and $\mathrm{t}{\overline{\mathrm{t}}} + \text{jets}$), as well as signal samples of gluino and squark pair production, are generated at leading order (LO) precision with the MadGraph  5 generator [30, 31] interfaced with pythia 8.2 [32] for fragmentation and parton showering. Up to four, three, or two additional partons are considered in the matrix element calculations for the generation of the $\text{V}+\text{jets}$ $(\text{V} = \text{Z, W})$, $\mathrm{t} {\overline{\mathrm{t}}} + \text {jets}$, and signal samples, respectively. Other background processes are also considered: $\mathrm{t}{\overline{\mathrm{t}}} \text {V} (\text{V} = \text{Z, W})$ samples are generated at LO precision with the MadGraph  5 generator, with up to two additional partons in the matrix element calculations, while single top samples are generated at next-to-leading order (NLO) precision with the MadGraph _aMC@NLO [30] or powheg  [33, 34] generators. Contributions from rarer processes such as diboson, triboson, and four top production, are found to be negligible. Standard model samples are simulated with a detailed Geant4  [35] based detector simulation and processed using the same chain of reconstruction programs as collision data, while the CMS fast simulation program [36] is used for the signal samples. The most precise available cross section calculations are used to normalize the simulated samples, corresponding most often to NLO or next-to-NLO accuracy [30, 33, 34, 37–40].

To improve on the MadGraph modeling of the multiplicity of additional jets from initial state radiation (ISR), MadGraph $\mathrm{t}{\overline{\mathrm {t}}}$ MC events are weighted based on the number of ISR jets ($N_{\mathrm {j}}^{\mathrm {ISR}}$) so as to make the jet multiplicity agree with data. The same reweighting procedure is applied to SUSY MC events. The weighting factors are obtained from a control region enriched in ${\mathrm {t}}{\overline{\mathrm {t}}}$, obtained by selecting events with two leptons and exactly two b-tagged jets, and vary between 0.92 for $N_{\mathrm {j}}^{\mathrm {ISR}}=1$ and 0.51 for $N_{\mathrm {j}}^{\mathrm {ISR}}\ge 6$. We take one half of the deviation from unity as the systematic uncertainty in these reweighting factors, to cover for differences between ${\mathrm {t}}{\overline{\mathrm{t}}}$ and SUSY production.

## Backgrounds

The backgrounds in jets-plus-$p_{\mathrm {T}} ^\text{miss}$ final states typically arise from three categories of SM processes:

- “lost lepton (LL)”, i.e., events with a lepton from a $\mathrm {W}$ decay where the lepton is either out of acceptance, not reconstructed, not identified, or not isolated.
  This background originates mostly from $\mathrm {W}\text {+jets}$ and $\mathrm {t}\overline{\mathrm {t}} \text {+jets}$ events, with smaller contributions from rarer processes such as diboson or $\mathrm {t}\overline{\mathrm {t}} \text{V} (\text {V}=\mathrm {Z},\mathrm {W})$ production.
- “irreducible”, i.e., $\mathrm {Z} \text {+jets}$ events, where the $\mathrm {Z}$ boson decays to neutrinos. This background is most similar to potential signals. It is a major background in nearly all search regions, its importance decreasing with increasing $N_{\mathrm {b}}$.
- “instrumental background”, i.e., mostly multijet events with no genuine $p_{\mathrm {T}} ^\text {miss}$. These events enter a search region due to either significant jet momentum mismeasurements, or sources of anomalous noise.

### Estimation of the background from events with leptonic $\mathrm {W}$ boson decays

Control regions with exactly one lepton candidate are selected using the same triggers and preselections used for the signal regions, with the exception of the lepton veto, which is inverted. Selected events are binned according to the same criteria as the search regions, and the background in each signal bin, $N^{\mathrm {SR}}_{\mathrm {LL}}$, is obtained from the number of events in the control region, $N^{\mathrm {CR}}_{1\ell }$, using transfer factors according to:

2 $$\begin{aligned}&N^{\mathrm {SR}}_{\mathrm {LL}} \left( H_{\mathrm {T}},N_{\mathrm {j}},N_{\mathrm {b}},M_{\mathrm {T2}} \right) \nonumber \\&\quad =N^{\mathrm {CR}}_{1\ell } \left( H_{\mathrm {T}},N_{\mathrm {j}},N_{\mathrm {b}},M_{\mathrm {T2}} \right) \nonumber \\&\qquad \times R^{0\ell /1\ell }_{\mathrm {MC}} \left( H_{\mathrm {T}},N_{\mathrm {j}},N_{\mathrm {b}},M_{\mathrm {T2}} \right) \, k \left( M_{\mathrm {T2}} \right) . \end{aligned}$$

The single-lepton control region typically has 1–2 times as many events as the corresponding signal region. The factor $R^{0\ell /1\ell }_{\mathrm {MC}} \left( H_{\mathrm {T}},N_{\mathrm {j}},N_{\mathrm {b}},M_{\mathrm {T2}} \right)$ accounts for lepton acceptance and efficiency and the expected contribution from the decay of $\mathrm {W}$ bosons to hadrons through an intermediate $\mathrm {\tau }$ lepton. It is obtained from MC simulation, and corrected for measured differences in lepton efficiencies between data and simulation.

**Fig. 1:**
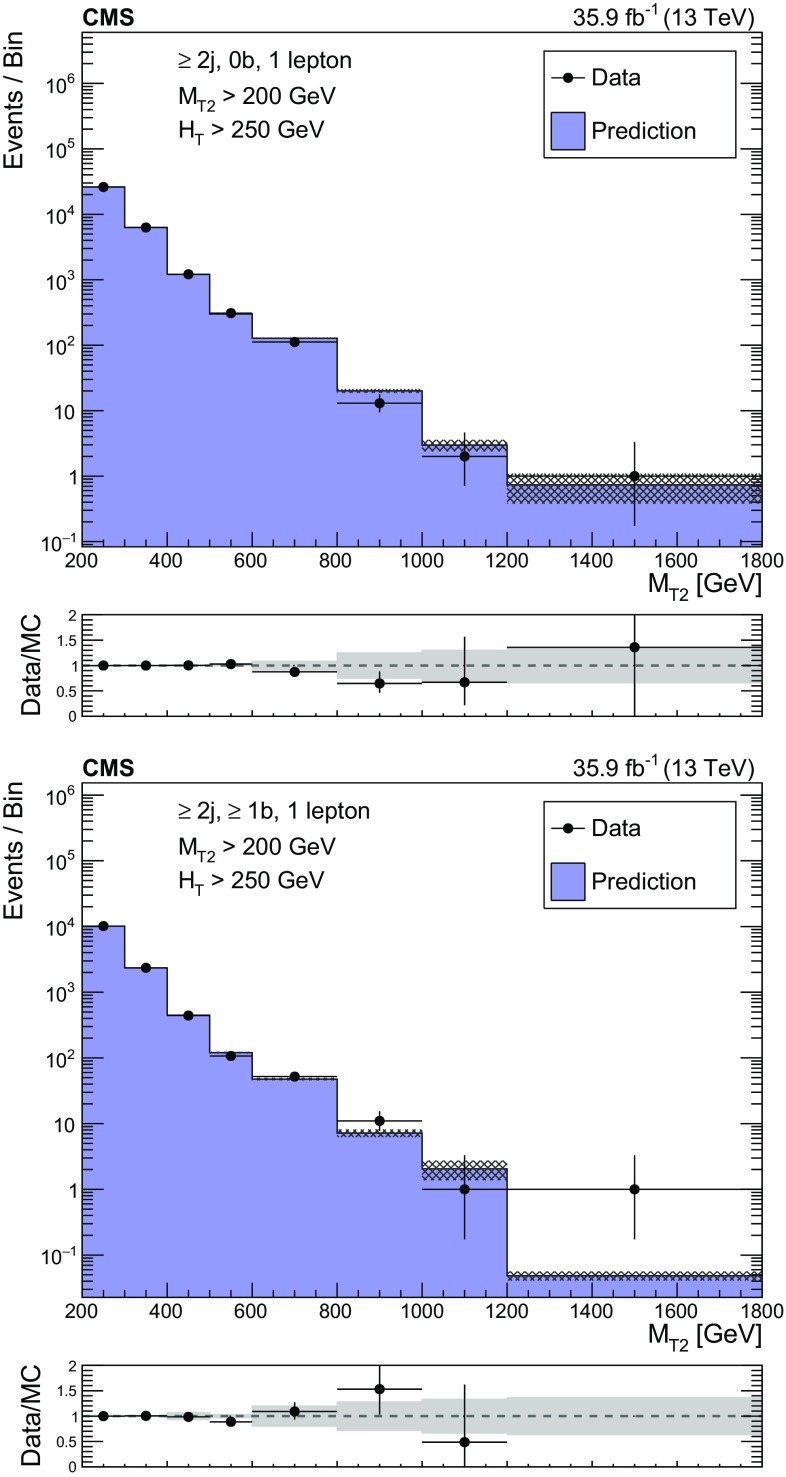
Distributions of the $M_{\mathrm {T2}}$ variable in data and simulation for the single-lepton control region selection, after normalizing the simulation to data in the control region bins of $H_{\mathrm {T}}$, $N_{\mathrm {j}}$, and $N_{\mathrm {b}}$ for events with no $\mathrm {b}$-tagged jets (upper), and events with at least one $\mathrm {b}$-tagged jet (lower). The hatched bands on the top panels show the MC statistical uncertainty, while the solid gray bands in the ratio plots show the systematic uncertainty in the $M_{\mathrm {T2}}$ shape

The factor $k\left( M_{\mathrm {T2}} \right)$ accounts for the distribution, in bins of $M_{\mathrm {T2}}$, of the estimated background in each topological region. It is obtained using both data and simulation as follows. In each topological region, the control region corresponding to the highest $M_{\mathrm {T2}}$ bin is successively combined with the next highest bin until the expected SM yield in combined bins is at least 50 events. When two or more control region bins are combined, the fraction of events expected to populate a particular $M_{\mathrm {T2}}$ bin, $k \left( M_{\mathrm {T2}} \right)$, is determined using the expectation from SM simulated samples, including all relevant processes. The modeling of $M_{\mathrm {T2}}$ is checked in data using single-lepton control samples enriched in events originating from either $\mathrm {W}\text {+jets}$ or $\mathrm {t}\overline{\mathrm {t}} \text {+jets}$, as shown in the upper and lower panels of Fig. 1, respectively. The predicted distributions in the comparison are obtained by summing all control regions after normalizing MC yields to data and distributing events among $M_{\mathrm {T2}}$ bins according to the expectation from simulation, as is done for the estimate of the lost-lepton background. For events with $N_{\mathrm {j}} =$ 1, a control region is defined for each bin of jet $p_{\mathrm {T}}$.

Uncertainties from the limited size of the control sample and from theoretical and experimental sources are evaluated and propagated to the final estimate. The dominant uncertainty in $R^{0\ell /1\ell }_{\mathrm {MC}} \left( H_{\mathrm {T}},N_{\mathrm {j}},N_{\mathrm {b}},M_{\mathrm {T2}} \right)$ arises from the modeling of the lepton efficiency (for electrons, muons, and hadronically-decaying tau leptons) and jet energy scale (JES) and is of order 15–20%. The uncertainty in the $M_{\mathrm {T2}}$ extrapolation, which is as large as 40%, arises primarily from the JES, the relative fractions of $\mathrm {W}\text {+jets}$ and $\mathrm {t}\overline{\mathrm {t}} \text {+jets}$, and variations of the renormalization and factorization scales assumed for their simulation. These and other uncertainties are similar to those in Ref. [6].

### Estimation of the background from $\mathrm {Z} (\nu \overline{\nu })+$jets

The $\mathrm {Z} \rightarrow \nu \overline{\nu }$ background is estimated from a dilepton control sample selected using triggers requiring two leptons. The trigger efficiency, measured with a data sample of events with large $H_{\mathrm {T}}$, is found to be greater than 97% in the selected kinematic range. To obtain a control sample enriched in $\mathrm {Z} \rightarrow \ell ^{+}\ell ^{-}$ events ($\ell = \mathrm {e},\mu$), we require that the leptons are of the same flavor, opposite charge, that the $p_{\mathrm {T}}$ of the leading and trailing leptons are at least 100 and 30 GeV, respectively, and that the invariant mass of the lepton pair is consistent with the mass of a $\mathrm {Z}$ boson within 20 GeV. After requiring that the $p_{\mathrm {T}}$ of the dilepton system is at least 200$\,\text {GeV}$, the preselection requirements are applied based on kinematic variables recalculated after removing the dilepton system from the event to replicate the $\mathrm {Z} \rightarrow \nu \overline{\nu }$ kinematics. For events with $N_{\mathrm {j}} = 1$, one control region is defined for each bin of jet $p_{\mathrm {T}}$. For events with at least two jets, the selected events are binned in $H_{\mathrm {T}}$, $N_{\mathrm {j}}$, and $N_{\mathrm {b}}$, but not in $M_{\mathrm {T2}}$, to increase the dilepton event yield in each control region.

The contribution to each control region from flavor-symmetric processes, most importantly $\mathrm {t}\overline{\mathrm {t}}$, is estimated using opposite-flavor (OF) $\mathrm {e}\mathrm {\mu }$ events obtained with the same selections as same-flavor (SF) $\mathrm {e}\mathrm {e}$ and $\mathrm {\mu }\mathrm {\mu }$ events. The background in each signal bin is then obtained using transfer factors according to:

3 $$\begin{aligned} N^{\mathrm {SR}}_{\mathrm {Z} \rightarrow \nu \overline{\nu }} \left( H_{\mathrm {T}},N_{\mathrm {j}},N_{\mathrm {b}},M_{\mathrm {T2}} \right)= & {} \Bigl [N^{\mathrm {CRSF}}_{\ell \ell } \left( H_{\mathrm {T}},N_{\mathrm {j}},N_{\mathrm {b}} \right) \nonumber \\&- N^{\mathrm {CROF}}_{\ell \ell } \left( H_{\mathrm {T}},N_{\mathrm {j}},N_{\mathrm {b}} \right) \, R^{\mathrm {SF}/\mathrm {OF}} \Bigr ]\nonumber \\&\times R^{\mathrm {Z} \rightarrow \nu \overline{\nu }/Z\rightarrow \ell ^{+}\ell ^{-}}_{\mathrm {MC}} \left( H_{\mathrm {T}},N_{\mathrm {j}},N_{\mathrm {b}} \right) \, k\left( M_{\mathrm {T2}} \right) . \end{aligned}$$

**Fig. 2:**
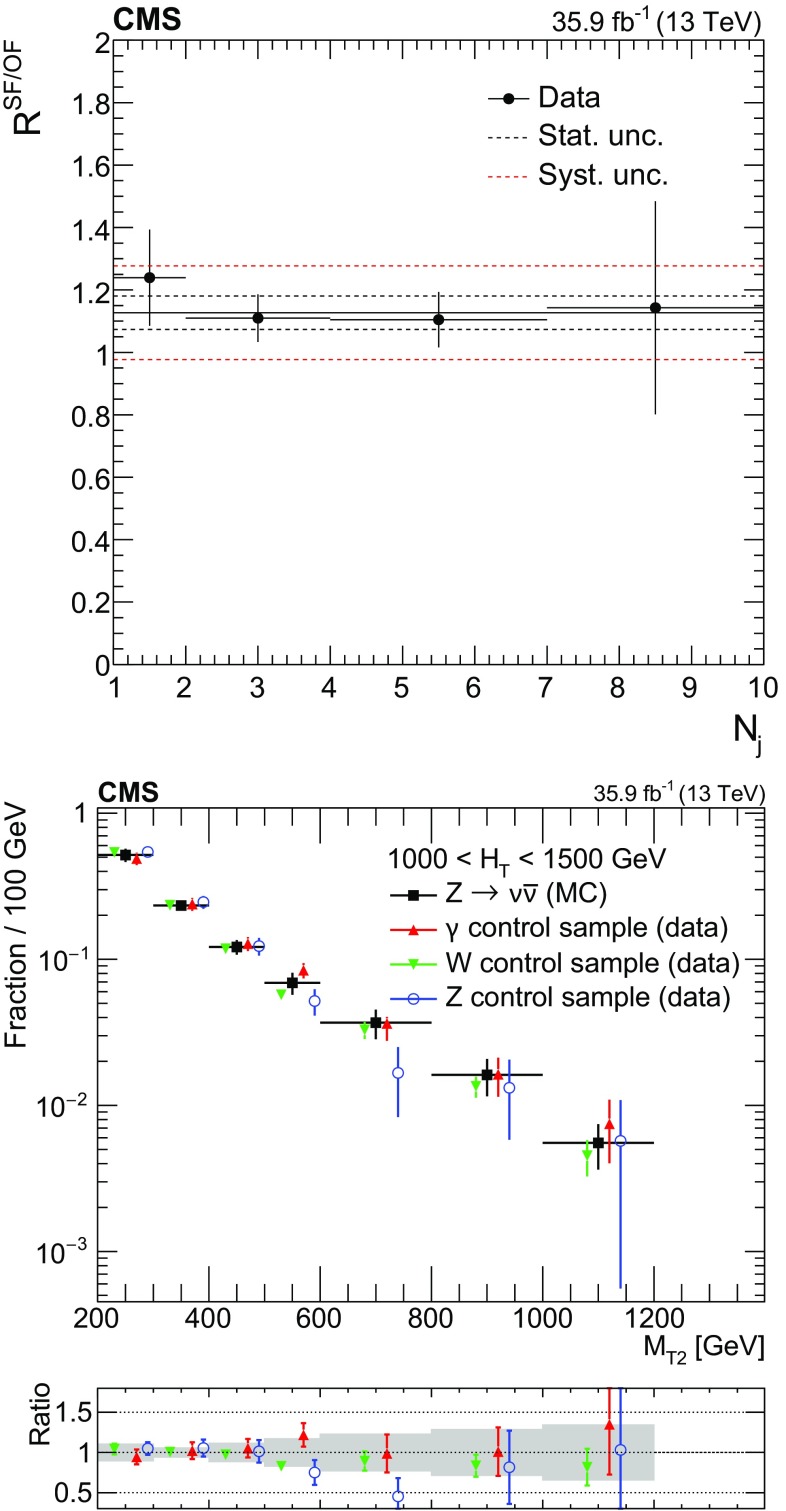
(Upper) Ratio $R^{\mathrm {SF}/\mathrm {OF}}$ in data as a function of $N_{\mathrm {j}}$. The solid black line enclosed by the red dashed lines corresponds to a value of $1.13\pm 0.15$ that is observed to be stable with respect to event kinematics, while the two dashed black lines denote the statistical uncertainty in the $R^{\mathrm {SF}/\mathrm {OF}}$ value. (Lower) The shape of the $M_{\mathrm {T2}}$ distribution in $\mathrm {Z} \rightarrow \nu \overline{\nu }$ simulation compared to shapes from $\gamma$, $\mathrm {W}$, and $\mathrm {Z}$ data control samples in a region with $1000<H_{\mathrm {T}} <1500\,\text {GeV}$ and $N_{\mathrm {j}} \ge 2$, inclusive in $N_{\mathrm {b}}$. The solid gray band on the ratio plot shows the systematic uncertainty in the $M_{\mathrm {T2}}$ shape

Here $N^{\mathrm {CRSF}}_{\ell \ell }$ and $N^{\mathrm {CROF}}_{\ell \ell }$ are the number of SF and OF events in the control region, while $R^{\mathrm {Z} \rightarrow \nu \overline{\nu }/\mathrm {Z} \rightarrow \ell ^{+}\ell ^{-}}_{\mathrm {MC}}$ and $k\left( M_{\mathrm {T2}} \right)$ are defined below. The factor $R^{\mathrm {SF}/\mathrm {OF}}$ accounts for the difference in acceptance and efficiency between SF and OF events. It is determined as the ratio of the number of SF events to OF events in a $\mathrm {t}\overline{\mathrm {t}}$ enriched control sample, obtained with the same selections as the $\mathrm {Z} \rightarrow \ell ^{+}\ell ^{-}$ sample, but inverting the requirements on the $p_{\mathrm {T}}$ and the invariant mass of the lepton pair. A measured value of $R^{\mathrm {SF}/\mathrm {OF}}=1.13\pm 0.15$ is observed to be stable with respect to event kinematics, and is applied in all regions. Figure 2 (left) shows $R^{\mathrm {SF}/\mathrm {OF}}$ measured as a function of the number of jets.

An estimate of the $\mathrm {Z} \rightarrow \nu \overline{\nu }$ background in each topological region is obtained from the corresponding dilepton control region via the factor $R^{\mathrm {Z} \rightarrow \nu \overline{\nu }/\mathrm {Z} \rightarrow \ell ^{+}\ell ^{-}}_{\mathrm {MC}}$, which accounts for the acceptance and efficiency to select the dilepton pair and the ratio of branching fractions for $\mathrm {Z} \rightarrow \ell ^{+}\ell ^{-}$ and $\mathrm {Z} \rightarrow \nu \overline{\nu }$ decays. This factor is obtained from simulation, including corrections for differences in the lepton efficiencies between data and simulation.

The factor $k\left( M_{\mathrm {T2}} \right)$ accounts for the distribution, in bins of $M_{\mathrm {T2}}$, of the estimated background in each topological region. This distribution is constructed using the $M_{\mathrm {T2}}$ shape from dilepton data and $\mathrm {Z} \rightarrow \nu \overline{\nu }$ simulation in each topological region. Studies with simulated samples indicate that the $M_{\mathrm {T2}}$ shape for $\mathrm {Z} \rightarrow \nu \overline{\nu }$ events is independent of $N_{\mathrm {b}}$ for a given $H_{\mathrm {T}}$ and $N_{\mathrm {j}}$ selection, and that the shape is also independent of the number of jets for $H_{\mathrm {T}} >1500\,\text {GeV}$. The MC modeling of $N_{\mathrm {b}}$ and $N_{\mathrm {j}}$ as well as of the $M_{\mathrm {T2}}$ shape in bins of $N_{\mathrm {j}}$ and $N_{\mathrm {b}}$ is validated in data, using a dilepton control sample. As a result, $M_{\mathrm {T2}}$ templates for topological regions differing only in $N_{\mathrm {b}}$ are combined, separately for data and simulation. For $H_{\mathrm {T}} >1500\,\text {GeV}$, only one $M_{\mathrm {T2}}$ template is constructed for data and one for simulation by combining all relevant topological regions.

Starting from the highest $M_{\mathrm {T2}}$ bin in each control region, we merge bins until the sum of the merged bins contains at least 50 expected events from simulation. The fraction of events in each uncombined bin is determined using the corresponding $M_{\mathrm {T2}}$ template from dilepton data, corrected by the ratio $R^{Z\rightarrow \nu \overline{\nu }/Z\rightarrow \ell ^{+}\ell ^{-}}_{\mathrm {MC}}$. The $M_{\mathrm {T2}}$ shape from simulation is used to distribute events among the combined bins, after normalizing the simulation to the data yield in the same group of bins.

The modeling of $M_{\mathrm {T2}}$ is validated in data using control samples enriched in $\gamma$, $\mathrm {W}\rightarrow \ell \nu$, and $\mathrm {Z} \rightarrow \ell ^{+}\ell ^{-}$ events in each bin of $H_{\mathrm {T}}$. The lower panel of Fig. 2 shows agreement between the $M_{\mathrm {T2}}$ distributions obtained from $\gamma$, $\mathrm {W}$, and $\mathrm {Z}$ data control samples with that from $\mathrm {Z} \rightarrow \nu \overline{\nu }$ simulation for events with $1000<H_{\mathrm {T}} <1500\,\text {GeV}$. In this comparison, the $\gamma$ sample is obtained by selecting events with $p_{\mathrm {T}} ^{\gamma }>180$ $\,\text {GeV}$ and is corrected for contributions from multijet events and $R^{\mathrm {Z}/\gamma }_{\mathrm {MC}}$, the $\mathrm {W}$ sample is corrected for $R^{\mathrm {Z}/\mathrm {W}}_{\mathrm {MC}}$, both the $\mathrm {W}$ and $\mathrm {Z}$ samples are corrected for contributions from top quark events, and the $\mathrm {Z}$ sample is further corrected for $R^{\mathrm {Z} \rightarrow \nu \overline{\nu }/\mathrm {Z} \rightarrow \ell ^{+}\ell ^{-}}_{\mathrm {MC}}$. Here $R^{\mathrm {Z}/\gamma }_{\mathrm {MC}}$ ($R^{\mathrm {Z}/\mathrm {W}}_{\mathrm {MC}}$) is the ratio of the $M_{\mathrm {T2}}$ distributions for $\mathrm {Z}$ boson and $\gamma$ ($\mathrm {W}$) boson events derived in simulation.

The largest uncertainty in the estimate of the invisible $\mathrm {Z}$ background in most regions results from the limited size of the dilepton control sample. This uncertainty, as well as all other relevant theoretical and experimental uncertainties, are evaluated and propagated to the final estimate. The dominant uncertainty in the ratio $R^{Z\rightarrow \nu \overline{\nu }/Z\rightarrow \ell ^{+}\ell ^{-}}_{\mathrm {MC}}$ is obtained from measured differences in lepton efficiency between data and simulation, and is about 5%. The uncertainty in the $k\left( M_{\mathrm {T2}} \right)$ factor arises from data statistics for uncombined bins, while for combined bins it is due to uncertainties in the JES and variations in the renormalization and factorization scales. These can result in effects as large as 40%.

### Estimation of the multijet background

For events with at least two jets, a multijet-enriched control region is obtained in each $H_{\mathrm {T}}$ bin by inverting the $\varDelta \phi _{\text {min}}$ requirement described in Sect. 3. Events are selected using $H_{\mathrm {T}}$ triggers, and the extrapolation from low- to high-$\varDelta \phi _{\text {min}}$ is based on the following ratio:

4 $$\begin{aligned} r_{\phi }(M_{\mathrm {T2}}) = N(\varDelta \phi _{\min } > 0.3) / N(\varDelta \phi _{\min } < 0.3). \end{aligned}$$

**Fig. 3:**
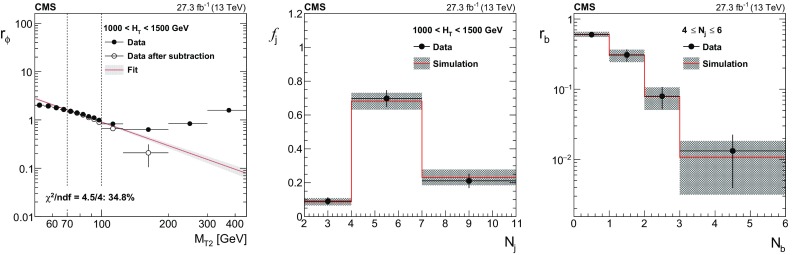
The ratio $r_{\phi }$ as a function of $M_{\mathrm {T2}}$ for $1000< H_{\mathrm {T}} < 1500$ $\,\text {GeV}$ (upper). The superimposed fit is performed to the open circle data points. The black points represent the data before subtracting non-multijet contributions using simulation. Data point uncertainties are statistical only. The red line and the grey band around it show the result of the fit to a power-law function performed in the window $70< M_{\mathrm {T2}} <100$ $\,\text {GeV}$ and the associated fit uncertainty. Values of $f_\mathrm {j}$, the fraction of events in bin $N_{\mathrm {j}}$, (middle) and $r_{\mathrm {b}}$, the fraction of events in bin $N_{\mathrm {j}}$ that fall in bin $N_{\mathrm {b}}$, (lower) are measured in data after requiring $\varDelta \phi _{\text {min}} < 0.3$ and $100< M_{\mathrm {T2}} < 200$ $\,\text {GeV}$. The hatched bands represent both statistical and systematic uncertainties

Studies with simulated samples show that the ratio can be described by a power law as $r_{\phi }(M_{\mathrm {T2}}) = a \, M_{\mathrm {T2}} ^{b}$. The parameters *a* and *b* are determined separately in each $H_{\mathrm {T}}$ bin by fitting $r_{\phi }$ in an $M_{\mathrm {T2}}$ sideband in data after subtracting non-multijet contributions using simulation. The sideband spans $M_{\mathrm {T2}}$ values of 60–100$\,\text {GeV}$ for events with $H_{\mathrm {T}}<$ 1000$\,\text {GeV}$, and 70–100$\,\text {GeV}$ for events with larger values of $H_{\mathrm {T}}$. The fit to the $r_{\phi }$ distribution in the 1000 $< H_{\mathrm {T}}<$ 1500$\,\text {GeV}$ region is shown in Fig. 3 (left). The inclusive multijet contribution in each signal region, $N^\mathrm {SR}_{\mathrm {j,b}}\left( M_{\mathrm {T2}} \right)$, is estimated using the ratio $r_{\phi }(M_{\mathrm {T2}})$ measured in the $M_{\mathrm {T2}}$ sideband and the number of events in the low-$\varDelta \phi _{\text {min}}$ control region, $N^\mathrm {CR}_{\mathrm {inc}}\left( M_{\mathrm {T2}} \right)$, according to

5 $$\begin{aligned} N^{\mathrm {SR}}_{\mathrm {j,b}}(M_{\mathrm {T2}}) = N^{\mathrm {CR}}_{\mathrm {inc}}\left( M_{\mathrm {T2}} \right) \, r_{\phi }(M_{\mathrm {T2}}) \, f_{\mathrm {j}} \left( H_{\mathrm {T}} \right) \, r_{\mathrm {b}} \left( N_{\mathrm {j}} \right) , \end{aligned}$$

where $f_\mathrm {j}$ is the fraction of multijet events in bin $N_{\mathrm {j}}$, and $r_{\mathrm {b}}$ is the fraction of events in bin $N_{\mathrm {j}}$ that are in bin $N_{\mathrm {b}}$. (Here, $N_{\mathrm {j}}$ denotes a jet multiplicity bin, and $N_{\mathrm {b}}$ denotes a $\mathrm {b}$ jet multiplicity bin within $N_{\mathrm {j}}$). The values of $f_\mathrm {j}$ and $r_{\mathrm {b}}$ are measured using events with $M_{\mathrm {T2}}$ between 100 and 200$\,\text {GeV}$ in the low $\varDelta \phi _{\text {min}}$ sideband, where $f_\mathrm {j}$ is measured separately in each $H_{\mathrm {T}}$ bin, while $r_{\mathrm {b}}$ is measured in bins of $N_{\mathrm {j}}$ integrated over $H_{\mathrm {T}}$, as $r_{\mathrm {b}}$ is found to be independent of the latter. Values of $f_\mathrm {j}$ and $r_{\mathrm {b}}$ measured in data are shown in Fig. 3 (center and right) compared to simulation.

The largest uncertainties in the estimate in most regions result from the statistical uncertainty in the fit and from the sensitivity of the $r_{\phi }$ value to variations in the fit window. These variations result in an uncertainty that increases with $M_{\mathrm {T2}}$ and ranges from 20–50%. The total uncertainty in the estimate is found to be of similar size as in Ref. [6], varying between 40–180% depending on the search region.

An estimate based on $r_{\phi }(M_{\mathrm {T2}})$ is not viable in the monojet search regions, which therefore require a different strategy. A control region is obtained by selecting events with a second jet with $30<p_{\mathrm {T}} < 60$ $\,\text {GeV}$ and inverting the $\varDelta \phi _{\text {min}}$ requirement. After subtracting non-multijet contributions using simulation, the data yield in the control region is taken as an estimate of the background in the corresponding monojet search region. Tests in simulation show the method provides a conservative estimate of the multijet background, which is less than 8% in all monojet search regions. In all monojet bins, a 50% uncertainty in the non-multijet subtraction is combined with the statistical uncertainty from the data yield in the control region with a second jet.

## Results

**Fig. 4:**
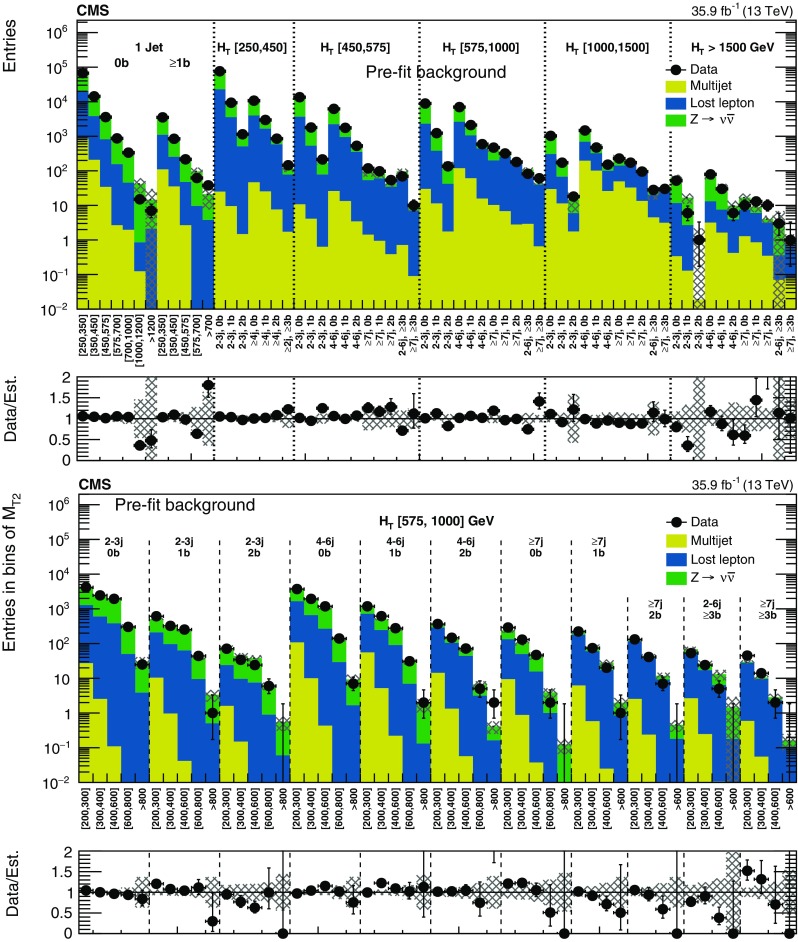
(Upper) Comparison of estimated (pre-fit) background and observed data events in each topological region. Hatched bands represent the full uncertainty in the background estimate. The results shown for $N_{\mathrm {j}} = 1$ correspond to the monojet search regions binned in jet $p_{\mathrm {T}}$, whereas for the multijet signal regions, the notations j, b indicate $N_{\mathrm {j}}$, $N_{\mathrm {b}}$ labeling. (Lower) Same for individual $M_{\mathrm {T2}}$ signal bins in the medium $H_{\mathrm {T}}$ region. On the *x*-axis, the $M_{\mathrm {T2}}$ binning is shown in units of $\,\text {GeV}$

The data yields in the search regions are statistically compatible with the estimated backgrounds from SM processes. A summary of the results of this search is shown in Fig. 4. Each bin in the upper panel corresponds to a single $H_{\mathrm {T}}$, $N_{\mathrm {j}}$, $N_{\mathrm {b}}$ topological region, integrated over $M_{\mathrm {T2}}$. The lower panel further breaks down the background estimates and observed data yields into $M_{\mathrm {T2}}$ bins for the region $575< H_{\mathrm {T}} <1000$ $\,\text {GeV}$. Distributions for the other $H_{\mathrm {T}}$ regions can be found in Appendix B. The background estimates and corresponding uncertainties shown in these plots rely exclusively on the inputs from control samples and simulation described in Sect. 4, and are referred to in the rest of the text as “pre-fit background” results.

To allow simpler reinterpretation, we also provide results for super signal regions, which cover subsets of the full analysis with simpler inclusive selections and that can be used to obtain approximate interpretations of this search. The definitions of these regions are given in Table 2, with the predicted and observed number of events and the 95% confidence level (CL) upper limit on the number of signal events contributing to each region. Limits are set using a modified frequentist approach, employing the CL$_{\mathrm {s}}$ criterion and relying on asymptotic approximations to calculate the distribution of the profile likelihood test-statistic used [41–44].

**Table 2:** Definitions of super signal regions, along with predictions, observed data, and the observed 95% CL upper limits on the number of signal events contributing to each region ($N_{95}^{\text {obs}}$). The limits are shown as a range corresponding to an assumed uncertainty in the signal acceptance of 0–15%. A dash in the selections means that no requirement is applied

Region	$N_{\mathrm {j}}$	$N_{\mathrm {b}}$	$H_{\mathrm {T}}$ ($\text {GeV}$ )	$M_{\mathrm {T2}}$ ($\text {GeV}$ )	Prediction	Data	$N_{95}^{\text {obs}}$
2j loose	$\ge$2	—	>1000	>1200	$38.9 \pm 11.2$	42	26.6–27.8
2j tight	$\ge$2	—	>1500	>1400	$2.9 \pm 1.3$	4	6.5–6.7
4j loose	$\ge$4	—	>1000	>1000	$19.4 \pm 5.8$	21	15.8–16.4
4j tight	$\ge$4	—	>1500	>1400	$2.1 \pm 0.9$	2	4.4–4.6
7j loose	$\ge$7	—	>1000	>600	$23.5^{+5.9}_{-5.6}$	27	18.0–18.7
7j tight	$\ge$7	—	>1500	>800	$3.1^{+1.7}_{-1.4}$	5	7.6–7.9
2b loose	$\ge$2	$\ge$2	>1000	>600	$12.9^{+2.9}_{-2.6}$	16	12.5–13.0
2b tight	$\ge$2	$\ge$2	>1500	>600	$5.1^{+2.7}_{-2.1}$	4	5.8–6.0
3b loose	$\ge$2	$\ge$3	>1000	>400	$8.4 \pm 1.8$	10	9.3–9.7
3b tight	$\ge$2	$\ge$3	>1500	>400	$2.0 \pm 0.6$	4	6.6–6.9
7j3b loose	$\ge$7	$\ge$3	>1000	>400	$5.1 \pm 1.5$	5	6.4–6.6
7j3b tight	$\ge$7	$\ge$3	>1500	>400	$0.9 \pm 0.5$	1	3.6–3.7

### Interpretation

The results of the search can be interpreted by performing a maximum likelihood fit to the data in the signal regions. The fit is carried out under either a background-only or a background+signal hypothesis. The uncertainties in the modeling of the backgrounds, summarized in Sect. 4, are inputs to the fitting procedure. The likelihood is constructed as the product of Poisson probability density functions, one for each signal region, with constraint terms that account for uncertainties in the background estimates and, if considered, the signal yields. The result of the background-only fit, denoted as “post-fit background”, is given in Appendix B. If the magnitude and correlation model of the uncertainties associated to the pre-fit estimates are properly assigned, and the data are found to be in agreement with the estimates, then the fit has the effect of constraining the background and reducing the associated uncertainties.

**Fig. 5:**
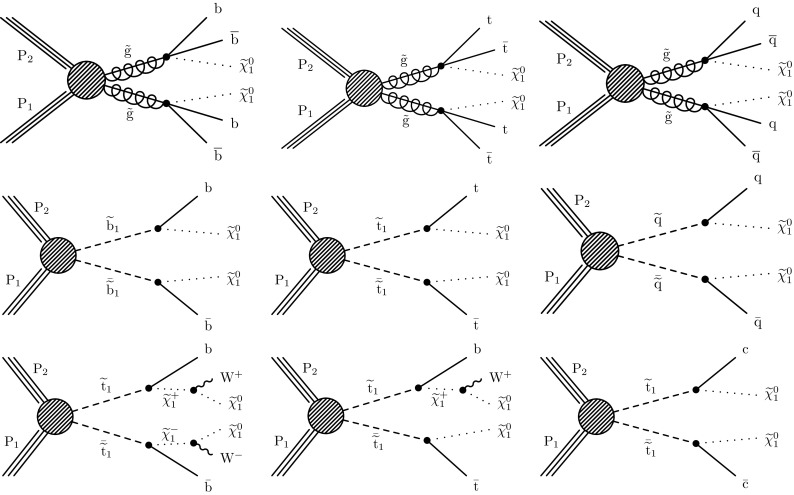
(Upper) Diagrams for the three scenarios of gluino-mediated bottom squark, top squark and light flavor squark production considered. (Middle) Diagrams for the direct production of bottom, top and light-flavor squark pairs. (Lower) Diagrams for three alternate scenarios of direct top squark production with different decay modes. For mixed decay scenarios, we assume a 50% branching fraction for each decay mode

The results of the search are used to constrain the simplified models of SUSY [45] shown in Fig. 5. For each scenario of gluino (squark) pair production, the simplified models assume that all SUSY particles other than the gluino (squark) and the lightest neutralino are too heavy to be produced directly, and that the gluino (squark) decays promptly. The models assume that each gluino (squark) decays with a 100% branching fraction into the decay products depicted in Fig. 5. For models where the decays of the two squarks differ, we assume a 50% branching fraction for each decay mode. For the scenario of top squark pair production, the polarization of the top quark is model dependent and is a function of the top-squark and neutralino mixing matrices. To remain agnostic to a particular model realization, events are generated without polarization. Signal cross sections are calculated at NLO+NLL order in $\alpha _{\mathrm {s}}$ [46–50].

Typical values of the uncertainties in the signal yield for the simplified models considered are listed in Table 3. The sources of uncertainties and the methods used to evaluate their effect on the interpretation are the same as those discussed in Ref. [6]. Uncertainties due to the luminosity [51], ISR and pileup modeling, and $\mathrm {b}$ tagging and lepton efficiencies are treated as correlated across search bins. Remaining uncertainties are taken as uncorrelated.

**Fig. 6:**
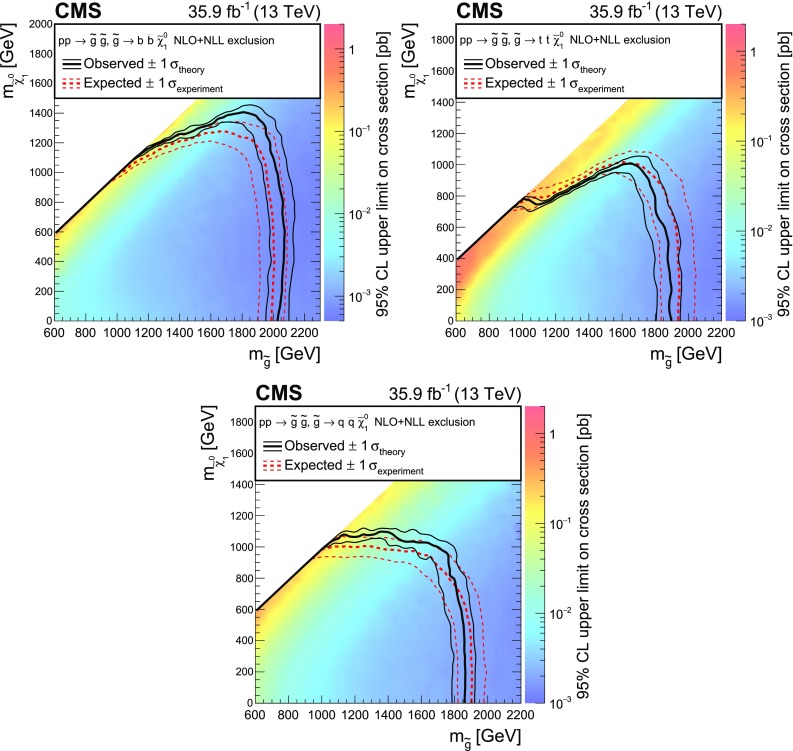
Exclusion limits at 95% CL for gluino-mediated bottom squark production (above left), gluino-mediated top squark production (above right), and gluino-mediated light-flavor ($\mathrm {u}$,$\mathrm {d}$,$\mathrm {s}$,$\mathrm {c}$) squark production (below). The area enclosed by the thick black curve represents the observed exclusion region, while the dashed red lines indicate the expected limits and their ±1 standard deviation ranges. The thin black lines show the effect of the theoretical uncertainties on the signal cross section

**Fig. 7:**
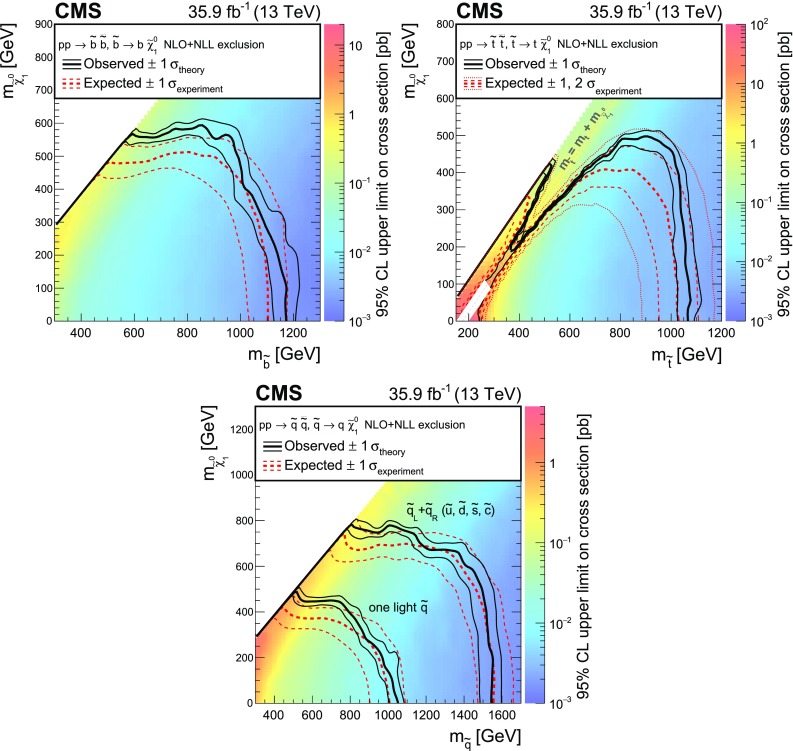
Exclusion limit at 95% CL for bottom squark pair production (above left), top squark pair production (above right), and light-flavor squark pair production (below). The area enclosed by the thick black curve represents the observed exclusion region, while the dashed red lines indicate the expected limits and their ±1 standard deviation ranges. For the top squark pair production plot, the ±2 standard deviation ranges are also shown. The thin black lines show the effect of the theoretical uncertainties on the signal cross section. The white diagonal band in the upper right plot corresponds to the region $|m_{\widetilde{\mathrm {t}}}-m_{\mathrm {t}}-m_{\widetilde{\chi }^{0}_{1}} |< 25\,\text {GeV}$ and small $m_{\widetilde{\chi }^{0}_{1}}$. Here the efficiency of the selection is a strong function of $m_{\widetilde{\mathrm {t}}}-m_{\widetilde{\chi }^{0}_{1}}$, and as a result the precise determination of the cross section upper limit is uncertain because of the finite granularity of the available MC samples in this region of the ($m_{\widetilde{\mathrm {t}}}, m_{\widetilde{\chi }^{0}_{1}}$) plane

**Fig. 8:**
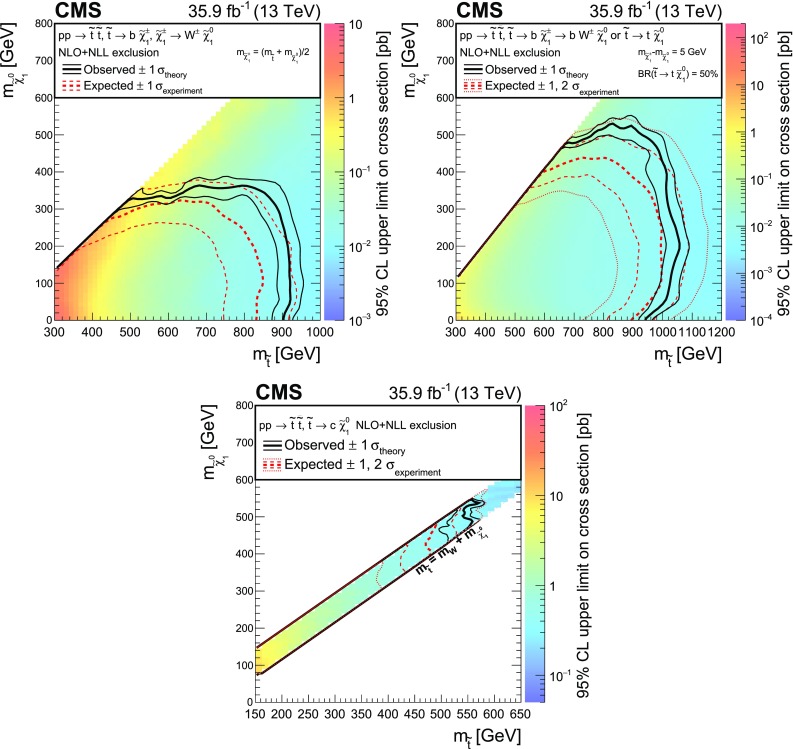
Exclusion limit at 95% CL for top squark pair production for different decay modes of the top squark. For the scenario where $\mathrm {p}\mathrm {p}\rightarrow \widetilde{\mathrm {t}} \overline{\widetilde{\mathrm {t}}} \rightarrow \mathrm {b} \overline{\mathrm {b}} \widetilde{\chi }^\pm _{1} \widetilde{\chi }^\mp _{1}$, $\widetilde{\chi }^\pm _{1} \rightarrow \mathrm {W}^{\pm } \widetilde{\chi }^{0}_{1}$ (above left), the mass of the chargino is chosen to be half way in between the masses of the top squark and the neutralino. A mixed decay scenario (above right), $\mathrm {p}\mathrm {p}\rightarrow \widetilde{\mathrm {t}} \overline{\widetilde{\mathrm {t}}}$ with equal branching fractions for the top squark decays $\widetilde{\mathrm {t}} \rightarrow \mathrm {t} \widetilde{\chi }^{0}_{1}$ and $\widetilde{\mathrm {t}} \rightarrow \mathrm {b} \widetilde{\chi }^{+}_{1}$, $\widetilde{\chi }^{+}_{1} \rightarrow \mathrm {W}^{*+}\widetilde{\chi }^{0}_{1}$, is also considered, with the chargino mass chosen such that $\varDelta m\left( \widetilde{\chi }^\pm _{1},\widetilde{\chi }^{0}_{1} \right) = 5\,\text {GeV}$. Finally, we also consider a compressed scenario (below) where $\mathrm {p}\mathrm {p}\rightarrow \widetilde{\mathrm {t}} \overline{\widetilde{\mathrm {t}}} \rightarrow \mathrm {c} \overline{\mathrm {c}} \widetilde{\chi }^{0}_{1} \widetilde{\chi }^{0}_{1}$. The area enclosed by the thick black curve represents the observed exclusion region, while the dashed red lines indicate the expected limits and their ±1 standard deviation ranges. The thin black lines show the effect of the theoretical uncertainties on the signal cross section

Figure 6 shows the exclusion limits at 95% CL for gluino-mediated bottom squark, top squark, and light-flavor squark production. Exclusion limits at 95% CL for the direct production of bottom, top, and light-flavor squark pairs are shown in Fig. 7. Direct production of top squarks for three alternate decay scenarios are also considered, and exclusion limits at 95% CL are shown in Fig. 8. Table 4 summarizes the limits on the masses of the SUSY particles excluded in the simplified model scenarios considered. These results extend the constraints on gluinos and squarks by about 300$\,\text {GeV}$ and on $\widetilde{\chi }^{0}_{1}$ by 200$\,\text {GeV}$ with respect to those in Ref. [6]. The largest differences between the observed and expected limits are found for scenarios of top squark pair production with moderate mass splittings and result from observed yields that are less than the expected background in topological regions with $H_{\mathrm {T}}$ between 575 and 1500 GeV, at least 7 jets, and either one or two b-tagged jets.

We note that the 95% CL upper limits on signal cross sections obtained using the most sensitive super signal regions of Table 2 are typically less stringent by a factor of $\sim$1.5–3 compared to those obtained in the fully-binned analysis. The full analysis performs better because of its larger signal acceptance and because it splits the events into bins with more favorable signal-to-background ratio.

**Table 3:** Typical values of the systematic uncertainties as evaluated for the simplified models of SUSY used in the context of this search. The high statistical uncertainty in the simulated signal sample corresponds to a small number of signal bins with low acceptance, which are typically not among the most sensitive signal bins to that model point.

Source	Typical values (%)
Integrated luminosity	2.5
Limited size of MC samples	1–100
Renormalization and factorization scales	5
ISR modeling	0–30
b Tagging efficiency, heavy flavors	0–40
b Tagging efficiency, light flavors	0–20
Lepton efficiency	0–20
Jet energy scale	5
Fast simulation $p_{\mathrm {T}} ^\text {miss}$ modeling	0–5
Fast simulation pileup modeling	4.6

**Table 4:** Summary of 95% CL observed exclusion limits on the masses of SUSY particles (sparticles) in different simplified model scenarios. The limit on the mass of the produced sparticle is quoted for a massless $\widetilde{\chi }^{0}_{1}$, while for the mass of the $\widetilde{\chi }^{0}_{1}$ we quote the highest limit that is obtained

Simplified	Limit on produced sparticle	Highest limit on the
model	mass ($\text {GeV}$ ) for $m_{\widetilde{\chi }^{0}_{1}}=0\,\text {GeV}$	$\widetilde{\chi }^{0}_{1}$ mass ($\text {GeV}$ )
Direct squark production:		
Bottom squark	1175	590
Top squark	1070	550
Single light squark	1050	475
Eight degenerate light squarks	1550	775
Gluino-mediated production:		
$\widetilde{\mathrm {g}} \rightarrow \mathrm {b} \overline{\mathrm {b}} \widetilde{\chi }^{0}_{1}$	2025	1400
$\widetilde{\mathrm {g}} \rightarrow \mathrm {t}\overline{\mathrm {t}} \widetilde{\chi }^{0}_{1}$	1900	1010
$\widetilde{\mathrm {g}} \rightarrow \mathrm {q} \overline{\mathrm {q}} \widetilde{\chi }^{0}_{1}$	1860	1100

## Summary

This paper presents the results of a search for new phenomena using events with jets and large $M_{\mathrm {T2}}$. Results are based on a 35.9$\,\text {fb}^\text {-1}$ data sample of proton–proton collisions at $\sqrt{s} =13\,\text {TeV}$ collected in 2016 with the CMS detector. No significant deviations from the standard model expectations are observed. The results are interpreted as limits on the production of new, massive colored particles in simplified models of supersymmetry. This search probes gluino masses up to 2025$\,\text {GeV}$ and $\widetilde{\chi }^{0}_{1}$ masses up to 1400$\,\text {GeV}$. Constraints are also obtained on the pair production of light-flavor, bottom, and top squarks, probing masses up to 1550, 1175, and 1070$\,\text {GeV}$, respectively, and $\widetilde{\chi }^{0}_{1}$ masses up to 775, 590, and 550$\,\text {GeV}$ in each scenario.

## Figures and Tables

**Fig. 9:**
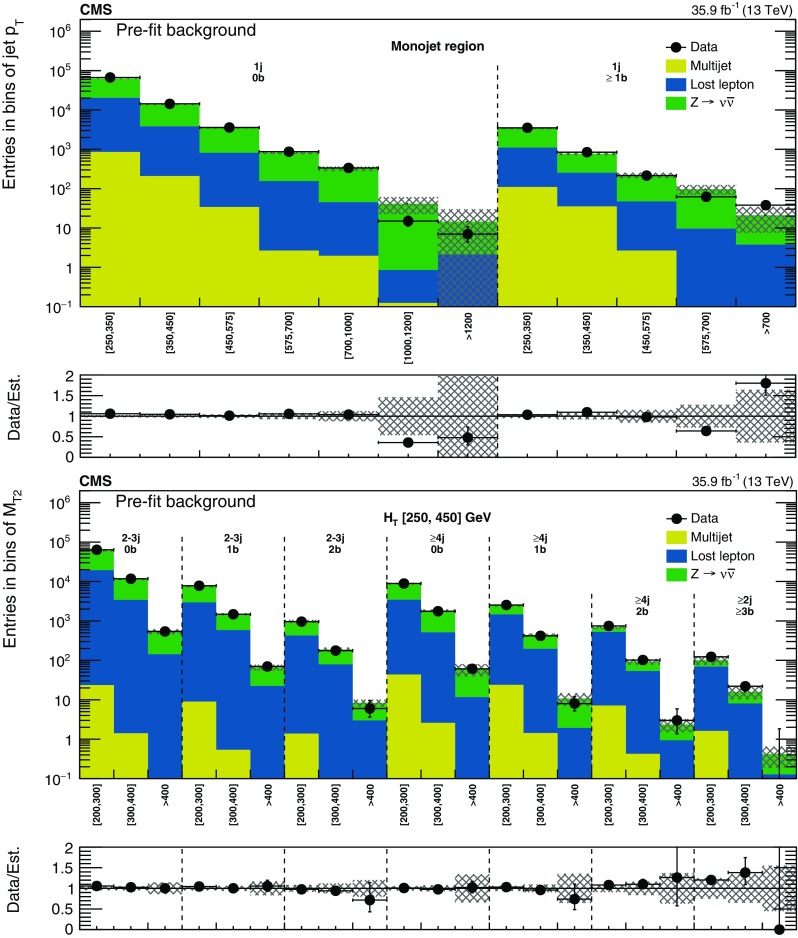
(Upper) Comparison of the estimated background and observed data events in each signal bin in the monojet region. On the *x*-axis, the $p_{\mathrm {T}} ^{\text {jet1}}$ binning is shown in units of $\,\text {GeV}$. Hatched bands represent the full uncertainty in the background estimate. (Lower) Same for the very low $H_{\mathrm {T}}$ region. On the *x*-axis, the $M_{\mathrm {T2}}$ binning is shown in units of $\,\text {GeV}$

**Fig. 10:**
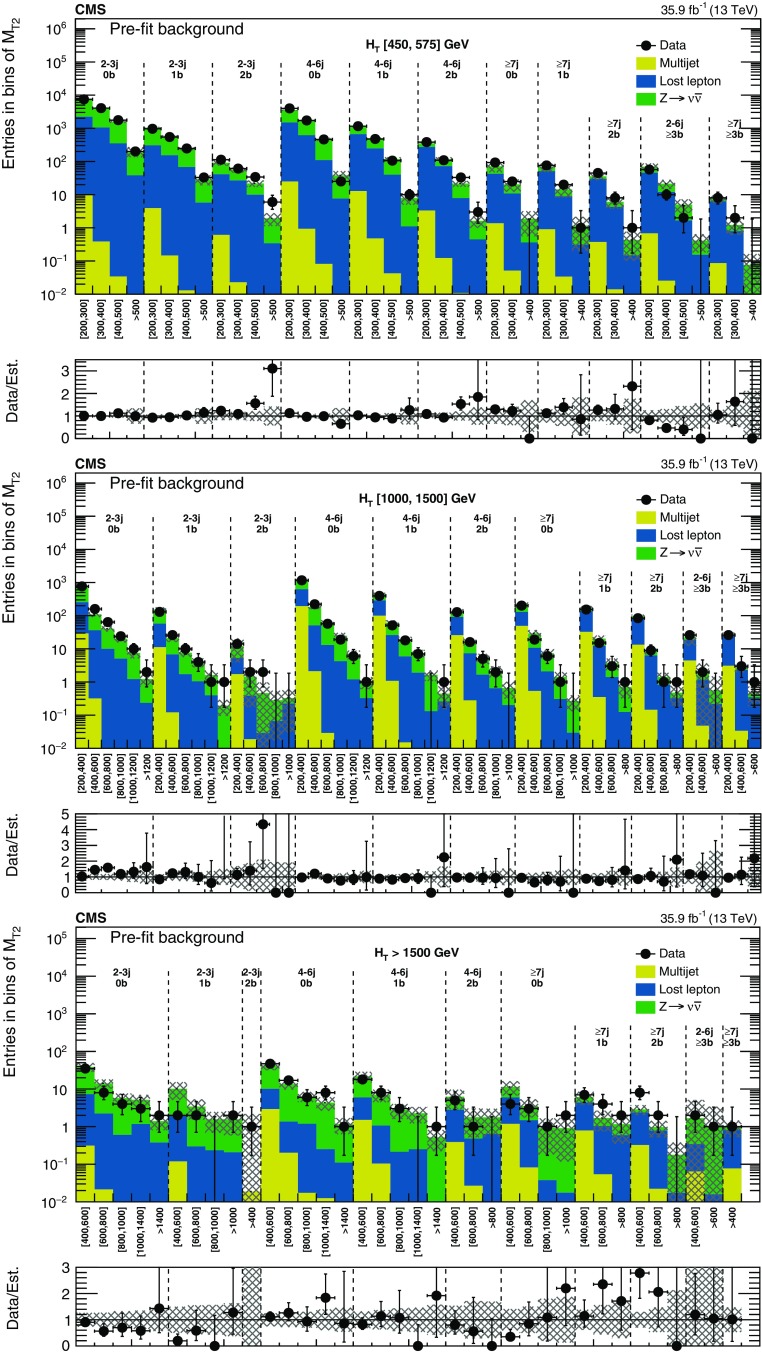
(Upper) Comparison of the estimated background and observed data events in each signal bin in the low-$H_{\mathrm {T}}$ region. Hatched bands represent the full uncertainty in the background estimate. Same for the high- (middle) and extreme- (lower) $H_{\mathrm {T}}$ regions. On the *x*-axis, the $M_{\mathrm {T2}}$ binning is shown in units of $\,\text {GeV}$. For the extreme-$H_{\mathrm {T}}$ region, the last bin is left empty for visualization purposes

**Fig. 11:**
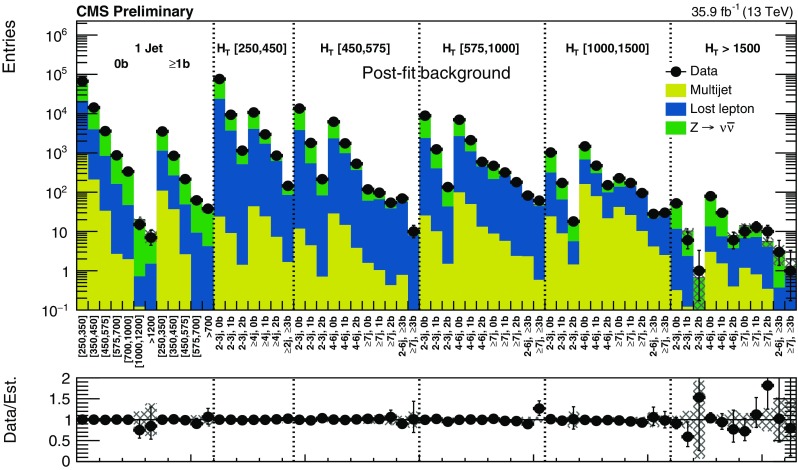
Comparison of post-fit background prediction and observed data events in each topological region. Hatched bands represent the post-fit uncertainty in the background prediction. For the monojet, on the *x*-axis the $p_{\mathrm {T}} ^{\text {jet1}}$ binning is shown in units of $\,\text {GeV}$, whereas for the multijet signal regions, the notations j, b indicate $N_{\mathrm {j}}$, $N_{\mathrm {b}}$ labeling

**Fig. 12:**
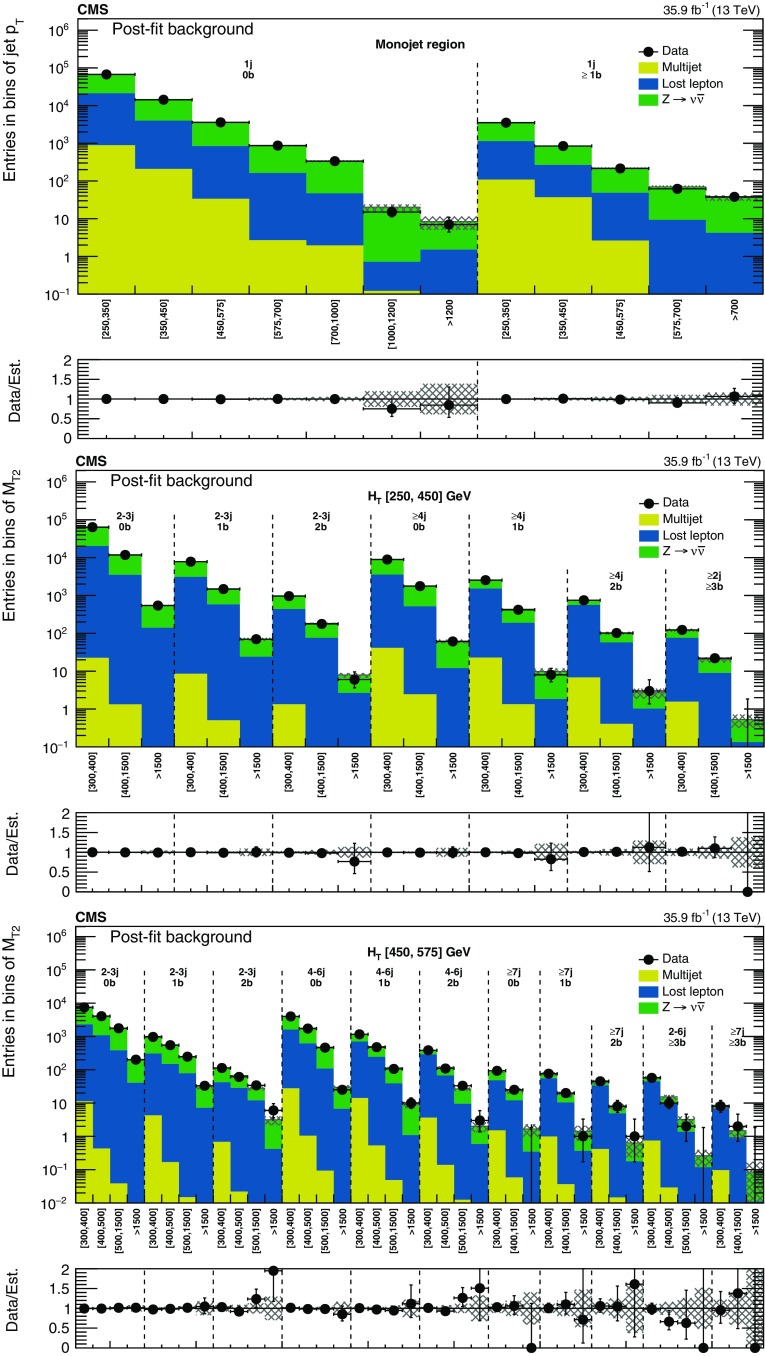
(Upper) Comparison of the post-fit background prediction and observed data events in each signal bin in the monojet region. On the *x*-axis, the $p_{\mathrm {T}} ^{\text {jet1}}$ binning is shown in units of $\,\text {GeV}$. (Middle) and (lower): Same for the very low and low-$H_{\mathrm {T}}$ region. On the *x*-axis, the $M_{\mathrm {T2}}$ binning is shown in units of $\,\text {GeV}$. The hatched bands represent the post-fit uncertainty in the background prediction

**Fig. 13:**
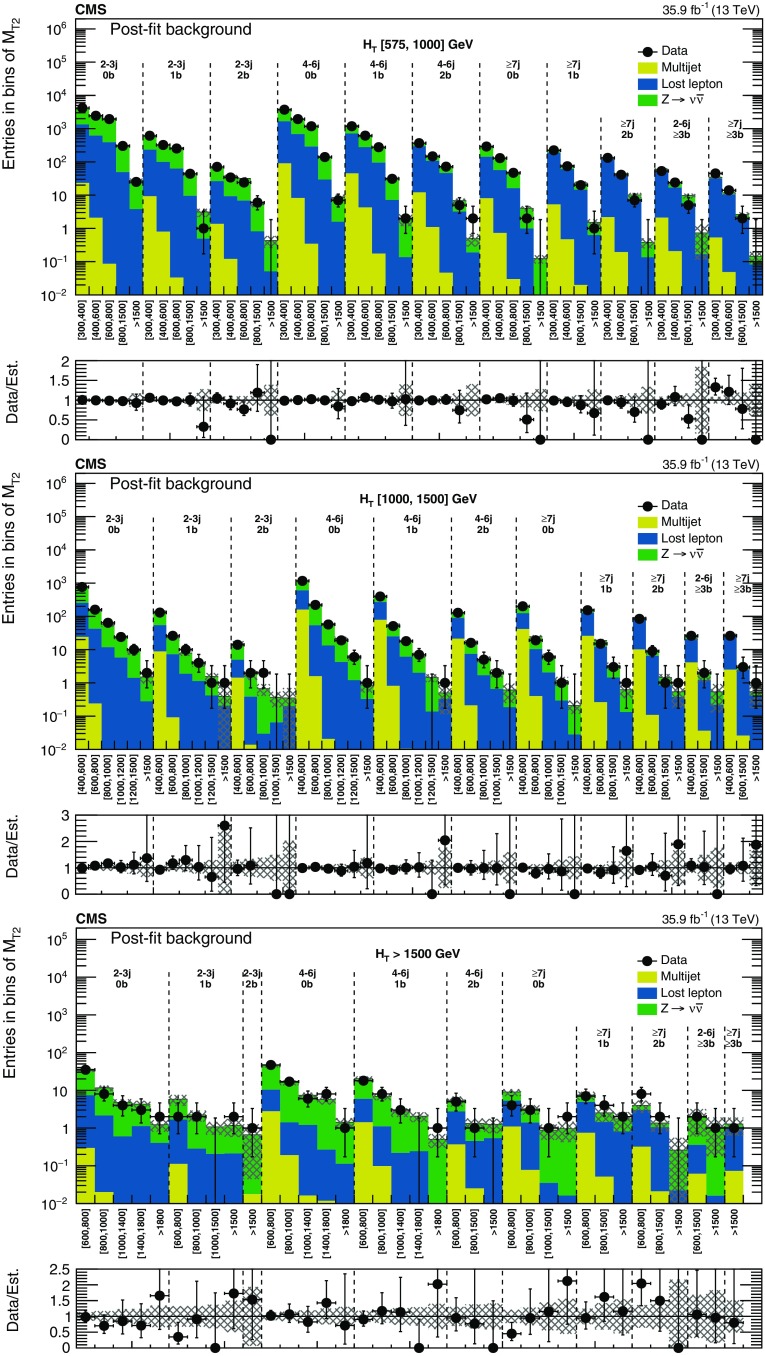
(Upper) Comparison of the post-fit background prediction and observed data events in each signal bin in the medium-$H_{\mathrm {T}}$ region. Same for the high- (middle) and extreme- (lower) $H_{\mathrm {T}}$ regions. On the *x*-axis, the $M_{\mathrm {T2}}$ binning is shown in units of $\,\text {GeV}$. The hatched bands represent the post-fit uncertainty in the background prediction. For the extreme-$H_{\mathrm {T}}$ region, the last bin is left empty for visualization purposes

**Fig. 14:**
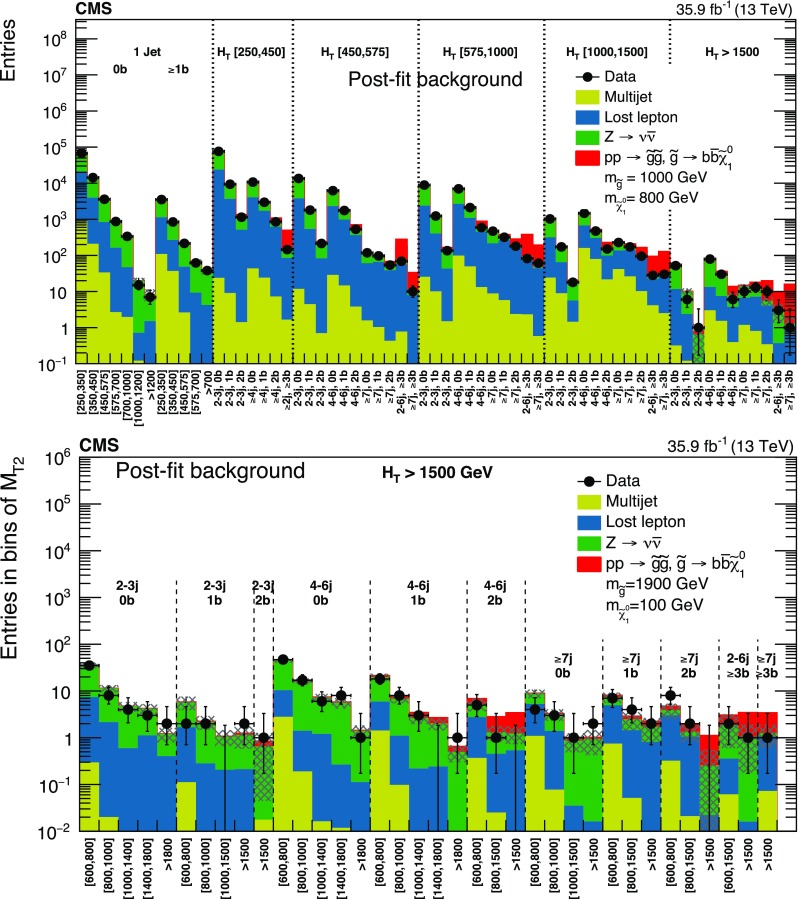
(Upper) The post-fit background prediction and observed data events in the analysis binning, for all topological regions with the expected yield for the signal model of gluino mediated bottom-squark production ($m_{\widetilde{\mathrm {g}}}=1000$ $\,\text {GeV}$, $m_{\widetilde{\chi }^{0}_{1}}=800$ $\,\text {GeV}$) stacked on top of the expected background. For the monojet regions, the $p_{\mathrm {T}} ^{\text {jet1}}$ binning is in units of $\,\text {GeV}$. (Lower) Same for the extreme-$H_{\mathrm {T}}$ region for the same signal with ($m_{\widetilde{\mathrm {g}}}=1900$ $\,\text {GeV}$, $m_{\widetilde{\chi }^{0}_{1}}=100$ $\,\text {GeV}$). On the *x*-axis, the $M_{\mathrm {T2}}$ binning is shown in units of $\,\text {GeV}$. The hatched bands represent the post-fit uncertainty in the background prediction. For the extreme-$H_{\mathrm {T}}$ region, the last bin is left empty for visualization purposes

## Definition of search regions

The 213 exclusive search regions are defined in Tables 5, 6 and 7.

**Table 5:** Summary of signal regions for the monojet selection

$N_{\mathrm {b}}$	Jet $p_{\mathrm {T}}$ binning ($\text {GeV}$ )
0	[250, 350, 450, 575, 700, 1000, 1200, $\infty$)
$\ge 1$	[250, 350, 450, 575, 700, $\infty$)

**Table 6:** The $M_{\mathrm {T2}}$ binning in each topological region of the multi-jet search regions, for the very low, low and medium $H_{\mathrm {T}}$ regions

$H_{\mathrm {T}}$ range ($\text {GeV}$ )	Jet multiplicities	$M_{\mathrm {T2}}$ binning ($\text {GeV}$ )
[ 250, 450 ]	$2-3$j, 0b	[ 200, 300, 400, $\infty$ )
	$2-3$j, 1b	[ 200, 300, 400, $\infty$ )
	$2-3$j, 2b	[ 200, 300, 400, $\infty$ )
	$\ge 4$j, 0b	[ 200, 300, 400, $\infty$ )
	$\ge 4$j, 1b	[ 200, 300, 400, $\infty$ )
	$\ge 4$j, 2b	[ 200, 300, 400, $\infty$ )
	$\ge 2$j, $\ge 3$b	[ 200, 300, 400, $\infty$ )
[ 450, 575 ]	$2-3$j, 0b	[ 200, 300, 400, 500, $\infty$ )
	$2-3$j, 1b	[ 200, 300, 400, 500, $\infty$ )
	$2-3$j, 2b	[ 200, 300, 400, 500, $\infty$ )
	$4-6$j, 0b	[ 200, 300, 400, 500, $\infty$ )
	$4-6$j, 1b	[ 200, 300, 400, 500, $\infty$ )
	$4-6$j, 2b	[ 200, 300, 400, 500, $\infty$ )
	$\ge 7$j, 0b	[ 200, 300, 400, $\infty$ )
	$\ge 7$j, 1b	[ 200, 300, 400, $\infty$ )
	$\ge 7$j, 2b	[ 200, 300, 400, $\infty$ )
	$2-6$j, $\ge 3$b	[ 200, 300, 400, 500, $\infty$ )
	$\ge 7$j, $\ge 3$b	[ 200, 300, 400, $\infty$ )
[ 575, 1000 ]	$2-3$j, 0b	[ 200, 300, 400, 600, 800, $\infty$ )
	$2-3$j, 1b	[ 200, 300, 400, 600, 800, $\infty$ )
	$2-3$j, 2b	[ 200, 300, 400, 600, 800, $\infty$ )
	$4-6$j, 0b	[ 200, 300, 400, 600, 800, $\infty$ )
	$4-6$j, 1b	[ 200, 300, 400, 600, 800, $\infty$ )
	$4-6$j, 2b	[ 200, 300, 400, 600, 800, $\infty$ )
	$\ge 7$j, 0b	[ 200, 300, 400, 600, 800, $\infty$ )
	$\ge 7$j, 1b	[ 200, 300, 400, 600, $\infty$ )
	$\ge 7$j, 2b	[ 200, 300, 400, 600, $\infty$ )
	$2-6$j, $\ge 3$b	[ 200, 300, 400, 600, $\infty$ )
	$\ge 7$j, $\ge 3$b	[ 200, 300, 400, 600, $\infty$ )

**Table 7:** The $M_{\mathrm {T2}}$ binning in each topological region of the multijet search regions, for the high- and extreme-$H_{\mathrm {T}}$ regions

$H_{\mathrm {T}}$ range ($\text {GeV}$ )	Jet multiplicities	$M_{\mathrm {T2}}$ binning ($\text {GeV}$ )
[ 1000, 1500 ]	$2-3$j, 0b	[ 200, 400, 600, 800, 1000, 1200, $\infty$ )
	$2-3$j, 1b	[ 200, 400, 600, 800, 1000, 1200, $\infty$ )
	$2-3$j, 2b	[ 200, 400, 600, 800, 1000, $\infty$ )
	$4-6$j, 0b	[ 200, 400, 600, 800, 1000, 1200, $\infty$ )
	$4-6$j, 1b	[ 200, 400, 600, 800, 1000, 1200, $\infty$ )
	$4-6$j, 2b	[ 200, 400, 600, 800, 1000, $\infty$ )
	$\ge 7$j, 0b	[ 200, 400, 600, 800, 1000, $\infty$ )
	$\ge 7$j, 1b	[ 200, 400, 600, 800, $\infty$ )
	$\ge 7$j, 2b	[ 200, 400, 600, 800, $\infty$ )
	$2-6$j, $\ge 3$b	[ 200, 400, 600, $\infty$ )
	$\ge 7$j, $\ge 3$b	[ 200, 400, 600, $\infty$ )
[ 1500, $\infty$ )	$2-3$j, 0b	[ 400, 600, 800, 1000, 1400, $\infty$ )
	$2-3$j, 1b	[ 400, 600, 800, 1000, $\infty$ )
	$2-3$j, 2b	[ 400, $\infty$ )
	$4-6$j, 0b	[ 400, 600, 800, 1000, 1400, $\infty$ )
	$4-6$j, 1b	[ 400, 600, 800, 1000, 1400, $\infty$ )
	$4-6$j, 2b	[ 400, 600, 800, $\infty$ )
	$\ge 7$j, 0b	[ 400, 600, 800, 1000, $\infty$ )
	$\ge 7$j, 1b	[ 400, 600, 800, $\infty$ )
	$\ge 7$j, 2b	[ 400, 600, 800, $\infty$ )
	$2-6$j, $\ge 3$b	[ 400, 600, $\infty$ )
	$\ge 7$j, $\ge 3$b	[ 400, $\infty$ )

## Detailed results

See Figs. 9, 10, 11, 12, 13 and 14 in appendix.
